# Spatiotemporally controlled O_2_ and singlet oxygen self-sufficient nanophotosensitizers enable the *in vivo* high-yield synthesis of drugs and efficient hypoxic tumor therapy†

**DOI:** 10.1039/d0sc02387f

**Published:** 2020-07-28

**Authors:** Suisui He, Siyu Lu, Sha Liu, Tianrong Li, Jieling Li, Shihao Sun, Meilin Liu, Kun Liang, Xu Fu, Fengjuan Chen, Genping Meng, Lang Zhang, Jun Hai, Baodui Wang

**Affiliations:** State Key Laboratory of Applied Organic Chemistry, Key Laboratory of Nonferrous Metal Chemistry and Resources Utilization of Gansu Province, Lanzhou University Lanzhou Gansu 730000 China haij@lzu.edu.cn wangbd@lzu.edu.cn; Laboratory of Emergency Medicine, Lanzhou University Second Hospital Lanzhou 730000 China; College of Chemistry and Molecular Engineering, Zhengzhou University Zhengzhou 450001 China; Institute of Anatomy and Histology & Embryology, School of Basic Medical Sciences, Lanzhou University Lanzhou 730000 China

## Abstract

Carrying out the *in vivo* syntheses of drugs toxic to tumors based on the specific features of the tumor microenvironment is critical for ensuring specific antitumor efficacy. However, achieving *in situ* high-yield synthetic toxic drugs from non-toxic agents and reducing their drug resistance in hypoxic tumors remain challenges. Herein we created a tumor-microenvironment-responsive porous Pt/Pt(iv) methylene blue coordination polymer nanoshuttle (Pt/PtMBCPNS) photosensitizer with spatiotemporally controlled O_2_ and singlet oxygen (^1^O_2_) self-sufficient for the *in vivo* high-yield synthesis of drugs and efficient hypoxic tumor therapy. After being endocytosed, the nanophotosensitizer as a cascade catalyst was observed to effectively catalyze the conversion of endogenous H_2_O_2_ to O_2_, and was hence found to play a dual role in the enhanced tumor therapy. PtMBCPNSs, upon being irradiated with red light, efficiently converted O_2_ into ^1^O_2_. Subsequently, ^1^O_2_ oxidized non-toxic 1,5-dihydroxynaphthalene to form the anticancer agent juglone with a high yield. In addition, O_2_ was found to be able to improve the hypoxic microenvironment without light irradiation, thus enhancing the antitumor efficacy of the produced drugs and reducing drug resistance. As a result, by enhancing the synergistic effect of the treatment, this nanophotosensitizer significantly inhibited the growth of tumors and avoided damage to normal tissues/organs. Collectively, this work highlights a robust nanoplatform with the spatiotemporally controlled *in vivo* high-yield synthesis of drugs and generation of O_2_ to help overcome the current limitations of chemical-based therapies against hypoxic tumors.

## Abstract 2

A porous photosensitizer displaying catalase-like activity and drug synthesis ability was synthesized for the synergistic chemo-photodynamic therapy, opening new promising ways for carrying out the precise cooperative treatment of hypoxic tumors.

## Graphical abstract

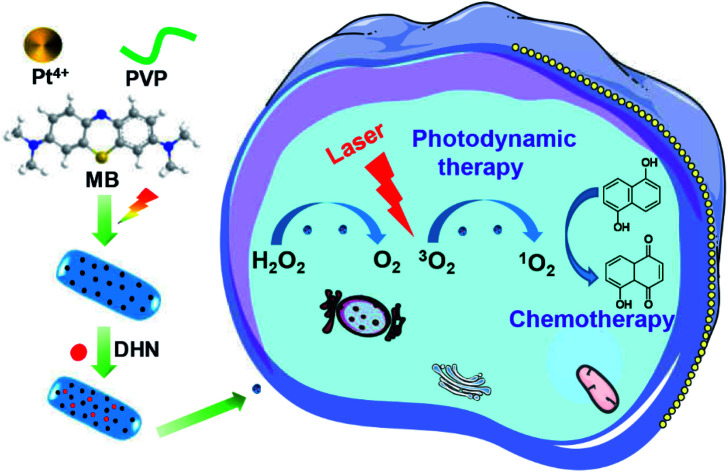

## 1. Introduction

Chemotherapy remains the main method used to treat cancer because chemotherapeutic agents often interfere with mitosis and are most toxic to rapidly dividing cells.^1^ However, chemotherapeutic drugs still have undesirable systemic side effects due to their low bioavailability and low specificity for cancer cells.^2,3^ In addition, some cancer cell lines also show resistance to chemotherapy, which reduces the efficiency of treatment.^4^ To minimize such systemic side effects, the tumor microenvironment (TME) has been extensively investigated in recent years, where cellular metabolism, the physical environment and biosynthetic intermediates are significantly different from those of normal tissues.^5^ Recently, drug release and diagnostic imaging techniques responding to the TME have been widely developed.^6–8^ However, the direct introduction of toxic chemotherapy drugs still leads to an undesirable distribution of these drugs in organisms, thereby destroying the normal tissues.^9,10^ To solve these problems, *in situ* syntheses of highly toxic anticancer drugs from non-toxic agents triggered by diverse artificially delivered nanoparticles in the TME have aroused great interest.^11–17^ Such a strategy would make the therapeutic process occur in only the tumor tissue without toxicity to normal tissues, so as to achieve high therapeutic efficacy while minimizing damage to normal tissues and/or organs. Unfortunately, the ultralow yields of toxic drugs have impeded their curative chemotherapy effect, limiting their further applications. In addition, worsened hypoxia reduces the cytotoxicity levels of drugs and increases drug resistance.^18^ Therefore, how to efficiently synthesize drugs and improve the issue of hypoxia at the tumor site is the main focus of chemotherapy research being carried out nowadays.

Naphthoquinone-based anticancer drugs such as juglone (5-hydroxy-1,4-naphthoquinone) constitute a class of successful Chinese herbal drugs and hold considerable promise for the chemotherapeutic treatment of various tumors including leukemia, melanoma, spontaneous breast cancer, pancreatic cancer, and gastric cancer.^19,20^ The anticancer activity of juglone involves inducing cell apoptosis through a mitochondrial-dependent pathway.^21^ Juglone is usually obtained by carrying out selective singlet oxygen (^1^O_2_) photooxidation of nontoxic 1,5-dihydroxynaphthalene (DHN).^22^ However, the low ^1^O_2_ generation efficiency leads to a low yield of juglone, which limits the localized large-scale synthesis. Therefore, it is highly desired to develop new photosensitizers to promote effective local ^1^O_2_ production and organic drug synthesis in the TME, avoiding the problems of delivery and distribution of toxic juglone.

Recently, coordination polymer (CP)-based photosensitizers with well-defined structures have shown great potential for O_2_ activation, because such structures can isolate photosensitizers (PSs) and largely prevent the aggregation-induced quenching (AIQ) of the photosensitizer molecules, thus improving the efficiency of ^1^O_2_ production.^23–26^ In addition, the formation of CP-based photosensitizers can also effectively reduce the single–triplet energy gap (Δ*E*_ST_) between the lowest-energy excited singlet state (S1) and lowest-energy excited triplet state (T1) of the photosensitizer.^27,28^ CP-based photosensitizers with lower Δ*E*_ST_ values would show improved intersystem crossing (ISC) rates and extended lifetimes in the triplet state. Both of these features are necessary to improve the transfer of energy from excited photosensitizer molecules to ground-state molecular oxygen (^3^O_2_) during the ^1^O_2_ production process.^29^ Moreover, O_2_ and ^1^O_2_ freely enter and diffuse out of the framework through the channels of porous photosensitizer-based CPs, and these free movements improve the usability of PSs.^30^ Nevertheless, such porous CPs with the aforementioned features have seldom been used as prodrug delivery systems for *in situ* photooxidation syntheses of toxic drugs against tumors based on the specific features of the TME.

**Scheme 1:**
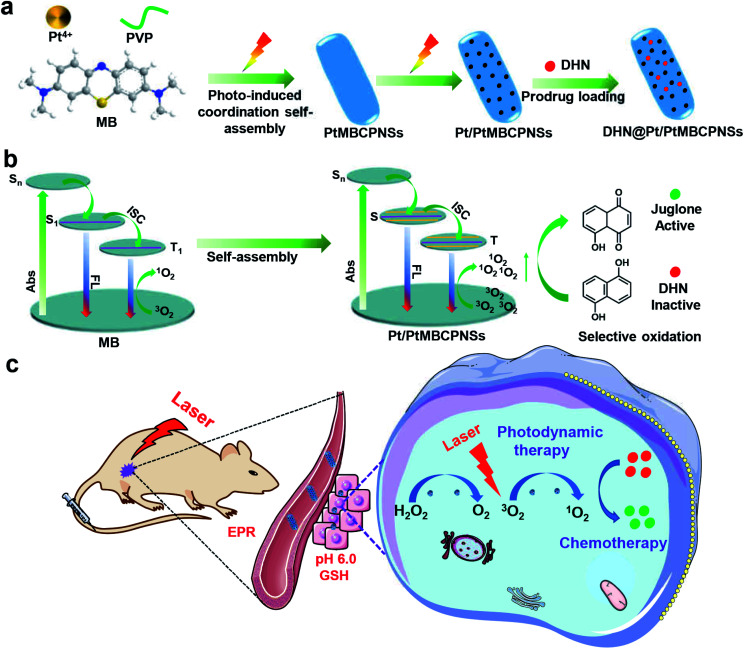
The synthesis and biological application of Pt/PtMBCPNSs. (a) Schematic illustrations of the preparation of Pt/PtMBCPNSs and loading of prodrugs. (b) Enhanced ^1^O_2_ production and selective oxidation of DHN (absorption is abbreviated as Abs, and fluorescence is abbreviated as FL). (c) The application of Pt/PtMBCPNSs in photodynamic therapy and chemotherapy.

In this study, we demonstrated a novel platinum(iv) methylene blue coordination polymer (PtMBCP)-engaged approach to synthesize size-switchable and porous Pt nanoparticle (NP)/PtMBCP nanoshuttles (Pt/PtMBCPNSs) through sequential topotactic conversions for *in vivo* drug synthesis and combination cancer therapy (Scheme 1). PtMBCP displaying a shuttle-like nanostructure was first prepared using a feasible photosynthesis method, and was employed as a template to adjust the morphology and size of the final product. Then, the pre-fabricated coordination polymer nanoshuttles were easily transformed into Pt/PtMBCPNSs upon being irradiated with visible light (Scheme 1a). Such Pt/PtMBCPNSs can improve energy transfer from excited photosensitizer molecules to O_2_ during the photodynamic process due to their relatively high ISC rate and long lifetime in the triplet state^29^ (Scheme 1b). The resulting nanoshuttles, as a sequential catalyst, can catalyze the conversion of H_2_O_2_ in the tumor area^31,32^ into O_2_ and effectively improve the production of ^1^O_2_ and selective photooxidation activity of DHN. Both *in vitro* and *in vivo* experiments established that ^1^O_2_ and juglone induced significant cytotoxicity in cancer cells and led to tumor regression under light irradiation (Scheme 1c). Thus, this superior Pt/PtMBCPNS system showed a high selectivity for cancer cells and a high ability to kill cells as a result of combined photodynamic therapy (PDT) and chemotherapy after systemic administration, reducing the side effects of traditional chemotherapy.

## 2. Results and discussion

### 2.1 Characterization of Pt/PtMBCPNSs

**Fig. 1:**
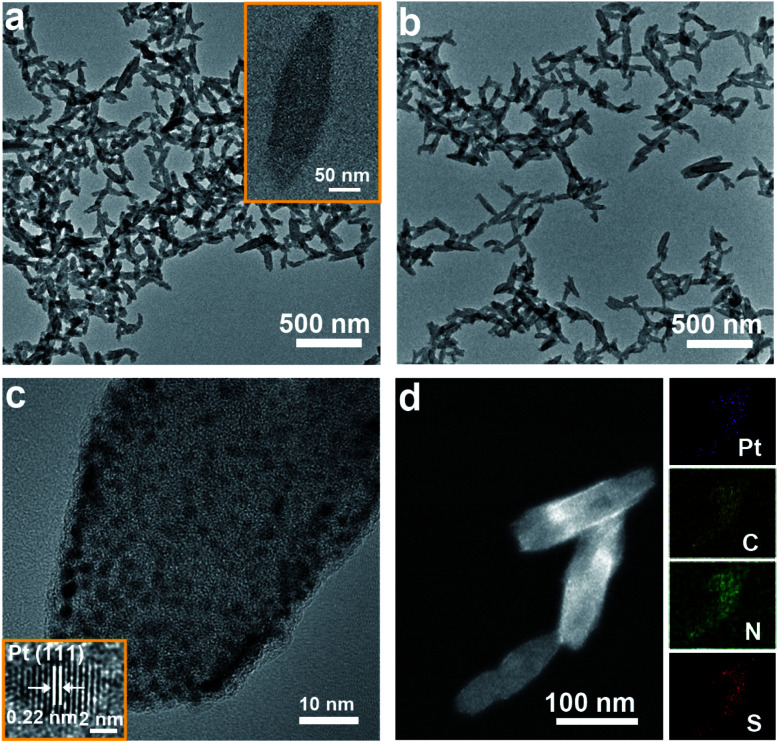
Characterizations of Pt/PtMBCPNSs. (a) A TEM image of PtMBCPNSs (with the inset showing a single PtMBCPNS). (b and c) TEM and HRTEM images of Pt/PtMBCPNSs. (d) A HAADF-STEM image and EDS elemental mapping of Pt/PtMBCPNSs.

Scheme 1a schematically shows the process used to synthesize the PtMBCPNSs and corresponding Pt/PtMBCPNSs. The acquired scanning electron microscopy (SEM) images revealed a shuttle-like structure for the formed PtMBCP (Fig. S1a†). Upon visible light irradiation for 30 minutes, the formed Pt/PtMBCP retained this shuttle-like structure (Fig. S1b†). Transmission electron microscopy (TEM) images showed that the PtMBCPNSs had an average length of 142.05 ± 4.45 nm and width of 46.9 ± 1.7 nm (Fig. 1a and S2†). After the formation of Pt/PtMBCPNSs (Fig. 1b and S1b†), Pt NPs with average dimensions of 1.8 ± 0.2 nm appeared (Fig. S3†) and were evenly distributed on the nanoshuttle (Fig. 1c). It can be seen from the acquired high-resolution TEM (HRTEM) image that the lattice spacing of the nanocrystal was 0.22 nm, a value matching the spacing of the Pt (111) crystal surface.^33^ The acquired HAADF-STEM images (Fig. 1d) and element mappings of Pt/PtMBCPNSs showed uniform distributions of S, C, N, and Pt in the nanoshuttles.

The acquired UV-Vis absorption spectra, displayed in Fig. S4,† showed the PtMBCPNSs and Pt/PtMBCPNSs exhibiting a broad absorption peak in the wavelength range 500–750 nm. However, the free MB only displayed a narrow absorption peak centered at 664 nm. The XRD data confirmed the amorphous natures of PtMBCPNSs and Pt/PtMBCPNSs (Fig. S5†). As shown in Fig. S6,† as compared to free MB, PtMBCPNSs and Pt/PtMBCPNSs yielded a new absorption peak at 414 cm^−1^, indicating a coordination between N of MB and Pt. The formations of PtMBCPNSs and Pt/PtMBCPNSs were further confirmed from the results of an X-ray photoelectron spectroscopy (XPS) investigation shown in Fig. S7.† Compared with MB, both PtMBCPNSs and Pt/PtMBCPNSs yielded new Pt 4f peaks (Fig. S7a†), revealing that PtMBCPNSs and Pt/PtMBCPNSs were successfully formed. Specifically, high-resolution spectra of PtMBCPNSs and Pt/PtMBCPNSs in the Pt 4f region (Fig. S7b†) both showed peaks belonging to Pt^4+^ species at 72.2 eV and 75.5 eV.^34^ Peaks belonging to the Pt^0^ species at 70.1 eV and 73.4 eV were observed in the spectrum of Pt/PtMBCPNSs but not in that of PtMBCPNSs.^35^ The acquired XPS spectra in the N 1s region showed a shift of an N 1s peak from 396.9 eV for MB to 397.2 eV for PtMBCPNSs and Pt/PtMBCPNSs (Fig. S7c†), indicative of a coordination of the N atom with Pt^4+^.^36^ Similarly, the acquired XPS spectra in the S 2p region showed a shift of S 2p peaks from 161.4 and 162.9 eV for MB to 161.5 and 163.0 eV for PtMBCPNSs and Pt/PtMBCPNSs (Fig. S7d†), revealing the coordination of the S atom with Pt^4+^.^37^

Application of the Barrett–Joyner–Halenda (BJH) method showed a mainly mesoporous structure for the Pt/PtMBCPNSs (Fig. S8a†), with an average pore size of 4.2 nm (Fig. S8b†). Like other mesoporous materials, Pt/PtMBCPNSs were expected to be able to adsorb prodrug molecules, and the adsorption of DHN by Pt/PtMBCPNSs was confirmed by the observation of an absorption peak at a wavelength of 298 nm in the corresponding UV-Vis absorption spectrum (Fig. S9†). The saturated loading capacity of DHN@Pt/PtMBCPNSs was 0.20 mg mg^−1^, illustrating that these nanoshuttles could be used as good carriers of DHN.

To reveal the process of the formation of Pt/PtMBCPNSs, time-dependent experiments were conducted and monitored using TEM (Fig. S10†). With only one minute of light illumination, a shuttle-shaped PtMBCP formed (Fig. S10b and f†). Significantly, after two minutes of light illumination, a small quantity of Pt NPs appeared on the nanoshuttle (Fig. S10c and g†). With the illumination time prolonged to five minutes, more Pt NPs dispersed onto the shuttle-like nanostructures (Fig. S10d and h†). Prolonging the illumination time to 30 minutes did not further change the composition of the shuttle-like nanostructures (Fig. S10e and f†).

### 2.2 Stability of Pt/PtMBCPNSs

To evaluate the stability of Pt/PtMBCPNSs under physiological conditions, samples were incubated with PBS at different pH levels (5.5, 6.0, 7.4), cell lysates, serum, and Dulbecco's Modified Eagle's Medium (DMEM) for 24 h, respectively. As shown in Fig. S11,† the shuttle-like morphology of the Pt/PtMBCPNSs was not destroyed in any of these media. And none of these incubations changed the positions of the XRD peaks (Fig. S12a†). Additionally, the FT-IR spectra of the Pt/PtMBCPNSs were almost the same as that of the original spectrum (Fig. S12b†). The above-described high stability levels of Pt/PtMBCPNSs in simulated biological environments guaranteed their good stability in the circulating blood before arriving to tumor tissue.

### 2.3 TME-triggered size change of Pt/PtMBCPNSs

**Fig. 2:**
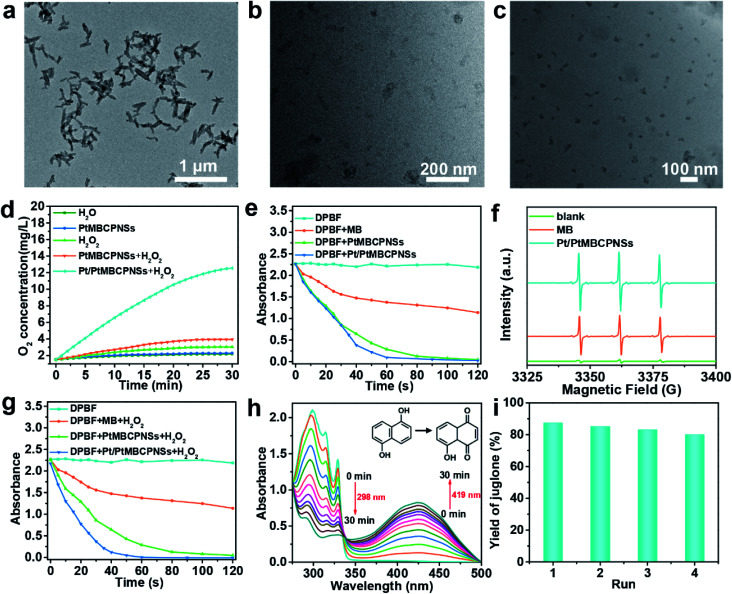
Evaluation of the ability of Pt/PtMBCPNSs to promote the generation of O_2_, ^1^O_2_, and juglone. (a–c) TEM images of Pt/PtMBCPNSs after they were incubated in PBS buffer solution under various conditions: (a) treatment for 24 h at pH 7.4, (b) treatment for 12 h at pH 6.0 with 10 mM GSH, and (c) treatment for 24 h at pH 6.0 with 10 mM GSH. (d) The amounts of O_2_ produced using various systems (H_2_O, Pt/PtMBCPNSs, H_2_O_2_, PtMBCPNSs + H_2_O_2_, and Pt/PtMBCPNSs + H_2_O_2_) over time. (e) Absorption values at 410 nm of DPBF in various solutions (alone, MB, PtMBCPNS and Pt/PtMBCPNS solutions, respectively) as a function of time of irradiation with light of a wavelength of 660 nm (100 mW cm^−2^). (f) EPR spectra of the samples after mixing a TEMP solution with MB and Pt/PtMBCPNSs under 660 nm-wavelength laser irradiation. (g) DPBF absorption values at 410 nm in MB, PtMBCPNS, and Pt/PtMBCPNS solutions with H_2_O_2_ as a function of time of irradiation with 660 nm-wavelength light (100 mW cm^−2^). (h) UV-Vis absorption spectra for DHN in PBS solution with Pt/PtMBCPNSs and subjected to photooxidation for various durations as indicated. (i) Yields of juglone for various numbers of cycles.

Generally speaking, once entering the tumor, nanovesicles become smaller as a result of stimulations by the corresponding components in the TME, which is very important to enhance penetration.^38^ As shown in Fig. 2a–c, compared with the Pt/PtMBCPNSs with an average length of 144.1 nm ± 4.45 nm and width of 50 ± 1.5 nm (Fig. S13a and b†) when placed in PBS buffer solution at pH 7.4 for 24 h (Fig. 2a), TEM images (Fig. 2b and c) indicated an obviously different morphology and dimensions for Pt/PtMBCPNSs placed in PBS buffer solution (pH 6.0) containing 10 mM glutathione (GSH)^39–41^ for 12 and 24 hours, for which the Pt/PtMBCPNSs transformed from the original shuttle-like shape to final spherical shapes with dimensions of 27.3 ± 1.3 nm (Fig. S13c†). The TEM, XRD, and XPS characterizations (Fig. S14–S16†) verified that the degradation products were small Pt/PtMBCP nanoparticles (Pt/PtMBCPNPs) (with the detailed analysis in the ESI†).

### 2.4 Evaluation of O_2_ and ^1^O_2_ generation

Hypoxia and acidic pH in the TME not only promote tumor invasion and metastasis but also result in tumor tolerance and resistance to a variety of treatments including immunotherapy.^42,43^ Recent research showed that the main way to improve the issue of hypoxia is to decompose excess H_2_O_2_ into O_2_ in the TME.^44–46^ In the current work, the O_2_ generation efficiencies of Pt/PtMBCPNSs were evaluated by using a portable meter for measuring dissolved oxygen (JPBJ-609L). As shown in Fig. 2d, O_2_ was rapidly produced upon adding Pt/PtMBCPNSs into the H_2_O_2_ solution. However, there was almost no O_2_ generation in the presence of PtMBCPNSs and the absence of Pt/PtMBCPNSs. Moreover, the Pt/PtMBCPNS catalyst showed good reusability and stability in four consecutive usage cycles (Fig. S17a and b†) under the experimental conditions. These results suggested that Pt/PtMBCPNSs could efficiently catalyze the decomposition of H_2_O_2_ to release O_2_, making it promising for improving the issue of hypoxia of the TME and the therapeutic effect.

The ^1^O_2_ production capability of Pt/PtMBCPNSs was measured by using 1,3-diphenylisobenzofuran (DPBF) as a probe. As shown in Fig. 2e, both Pt/PtMBCPNSs and PtMBCPNSs showed much more effective ^1^O_2_ generation than did free MB and DPBF alone upon being irradiated with a 660 nm-wavelength laser (Fig. S18a–d†). The type of free radical produced was further determined by performing electron paramagnetic resonance (EPR) experiments with 2,2,6,6-tetramethyl-4-piperidone (TEMP) as the specific spin trap reagent due to its ability to trap ^1^O_2_ and hence afford the stable nitroxide radical. As shown in Fig. 2f, ^1^O_2_ was the unique product of the reaction catalyzed by Pt/PtMBCPNSs, PtMBCPNSs and MB, and Pt/PtMBCPNSs showed a higher ^1^O_2_ production efficiency than did free MB. Such a high ^1^O_2_ production efficiency might stem from the increase of the number of energy transition channels and enhanced ISC after the formation of porous coordination polymers, consistent with porphyrinic-based MOF systems.^27,28^ To simulate the TME, we next investigated the capability of Pt/PtMBCPNSs to produce ^1^O_2_ in the presence of H_2_O_2_. As shown in Fig. 2g and S19,† Pt/PtMBCPNSs yielded an enhanced ^1^O_2_ generation rate. This result was due to the generation of ^1^O_2_ from H_2_O_2_ based on the cascade reaction equation 2H_2_O_2_ → 2H_2_O + O_2_ and O_2_ → ^1^O_2_. In contrast, free MB and PtMBCPNSs did not catalyze the decomposition of H_2_O_2_ to produce O_2_, as no increase in ^1^O_2_ generation rate was found. All of these results indicated that Pt/PtMBCPNSs can not only improve the hypoxic microenvironment of the cancer cell (*i.e.*, increase the level of oxygen) but also produce highly toxic ^1^O_2_ for the PDT.

### 2.5 Photooxidation of DHN

Previous studies have shown that ^1^O_2_ is crucial for highly selective photooxidation of DHN, and the product juglone is widely used as an antitumor drug.^47,48^ Due to its high selectivity and efficiency for ^1^O_2_ production, we used Pt/PtMBCPNSs to catalyze the oxidation of DHN to produce juglone. Its production was further confirmed from the results of a ^1^H NMR analysis (Fig. S20†). As shown in Fig. 2h, under the irradiation of a 660 nm-wavelength laser (100 mW cm^−2^), the intensity of the absorption at a wavelength of 298 nm, corresponding to DHN, decreased, and that at 419 nm, corresponding to juglone, increased in the PBS buffer (pH 6.0). The DHN photooxidation reaction rate constant (*k*) was 0.041 min^−1^ and the yield of juglone reached 87.23% (Fig. 2i), higher than that of other photooxidation systems.^22^

In the control experiment without Pt/PtMBCPNSs after irradiation for 30 min, hardly any change in absorption intensity of DHN was observed in the UV-Vis spectrum (Fig. S21†), and the amount of juglone produced was negligible. Furthermore, the catalyst showed good reusability (Fig. 2i) and stability under the experimental conditions, as confirmed by relative activity measurements and inspection of an SEM image of the cycled catalysts (Fig. S22†). Also, as the juglone was formed, the fluorescence of DHN disappeared (Fig. S23†). Additionally, other similar substrates, such as 1-naphthol and 1,6-DHN, were also oxidized to form the corresponding naphthaleneone substances with good yields (Fig. S24†).

### 2.6 Cellular uptake of DHN@Pt/PtMBCPNSs

Encouraged by the excellent O_2_-, ROS-, and chemical drug-producing abilities of DHN@Pt/PtMBCPNSs, we further studied their PDT and chemotherapy efficacies *in vitro*. To evaluate the efficiency of DHN@Pt/PtMBCPNSs at releasing DHN, samples of the resulting nanocapsule in a dialysis bag were placed in phosphate buffer solutions, one at pH 7.4 or the other at pH 6.0. As shown in Fig. S25,† up to 82% of the DHN was released from the DHN@Pt/PtMBCPNS nanocapsule in the presence of GSH at pH 6.0 after 36 hours of treatment, while only 30.5% of the DHN was released in the absence of GSH at pH 6.0, and 14.8% in the absence of GSH at pH 7.4. The increased percentage of the drug released from the DHN@Pt/PtMBCPNS nanocapsule was due to the degradation of Pt/PtMBCPNSs in GSH; this degradation was confirmed from TEM images (Fig. 2b and c). Under the excitation of 620 nm, the Pt/PtMBCPNSs showed red fluorescence emission (Fig. S26†). Therefore, we used confocal laser scanning microscopy (CLSM) to explore the intracellular performance of the DHN@Pt/PtMBCPNSs, specifically in HeLa cells. As shown in Fig. S27,† HeLa cells incubated with DHN@Pt/PtMBCPNSs for 2 h showed bright red fluorescence, indicating that the DHN@Pt/PtMBCPNSs could be endocytosed effectively.

**Fig. 3:**
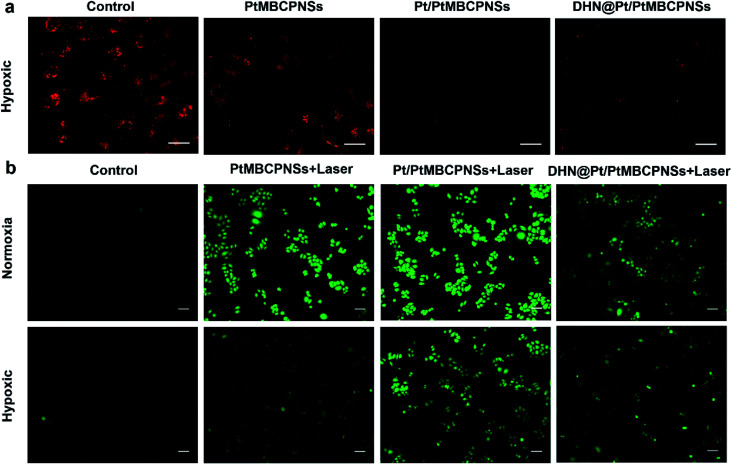
(a) CLSM images of intracellular O_2_ generated from HeLa cells after the cells were incubated with RDPP for 4 h under various treatment conditions: saline, PtMBCPNSs, Pt/PtMBCPNSs, and DHN@Pt/PtMBCPNSs under hypoxic conditions. (Scale bar = 40 μm.) (b) CLSM images of ROS produced in HeLa cells treated with Pt/PtMBCPNSs in the dark, PtMBCPNSs + laser (100 mW cm^−2^, 10 min), Pt/PtMBCPNSs + laser (100 mW cm^−2^, 10 min), and DHN@Pt/PtMBCPNSs + laser (100 mW cm^−2^, 10 min) under normoxic and hypoxic conditions. (Scale bar = 20 μm.)

The O_2_ production capacity of DHN@Pt/PtMBCPNSs in hypoxic HeLa cells was tracked by carrying out fluorescence imaging of hypoxia probes. Here, [Ru(dpp)_3_]Cl_2_ (RDPP) was used as a probe for intracellular hypoxia under a hypoxic atmosphere (1% O_2_, 5% CO_2_, and 94% N_2_). As shown in Fig. 3a, strong red fluorescence appeared in the saline-treated cells and PtMBCPNS-treated cells, indicating a hypoxic state. However, HeLa cells treated with the Pt/PtMBCPNS and DHN@Pt/PtMBCPNS groups showed weaker fluorescence levels, implying that Pt/PtMBCPNSs could catalyze the conversion of excess H_2_O_2_ in cancer cells to O_2_, thereby alleviating the intracellular hypoxia.

To confirm that DHN@Pt/PtMBCPNSs could produce ^1^O_2_ in cancer cells, we selected 2′,7′-dichlorofluorescein diacetate (DCFH-DA) as an intracellular ROS sensor since DCFH-DA can be oxidized by ROS to generate green fluorescent dichlorofluorescein (DCFDA). After irradiating the cancer cells for 10 minutes, a bright green light from DCFDA was observed, confirming that PtMBCPNSs, Pt/PtMBCPNSs, and DHN@Pt/PtMBCPNSs could produce ^1^O_2_ in the cancer cells under normoxic conditions (Fig. 3b). Under hypoxic conditions, the ^1^O_2_ levels of Pt/PtMBCPNS- as well as DHN@Pt/PtMBCPNS-treated cancer cells showed little change in comparison with those under normoxic conditions, whereas the ^1^O_2_ level of PtMBCPNS-treated cancer cells was much lower than that under normoxic conditions. Furthermore, the emission brightness indicated that the quantity of ^1^O_2_ generated by Pt/PtMBCPNSs in cancer cells was significantly higher than that generated by DHN@Pt/PtMBCPNSs, a result attributed to some of the ^1^O_2_ having been consumed for the oxidation of DHN to the anticancer juglone drug (Fig. S28†). The *in situ* generation of juglone in HeLa cells was further confirmed by inspecting CLSM images (Fig. S29†). As shown in Fig. S29a,† HeLa cells incubated with DHN@Pt/PtMBCPNSs showed bright blue fluorescence in the dark, which stemmed from DHN. However, the blue fluorescence in HeLa cells was significantly decreased upon 660 nm-wavelength laser irradiation (Fig. S29b†), indicating that the DHN was oxidized to form a non-fluorescent juglone.

### 2.7 *In vitro* cytotoxicity and synergistic PDT and chemotherapy performances

**Fig. 4:**
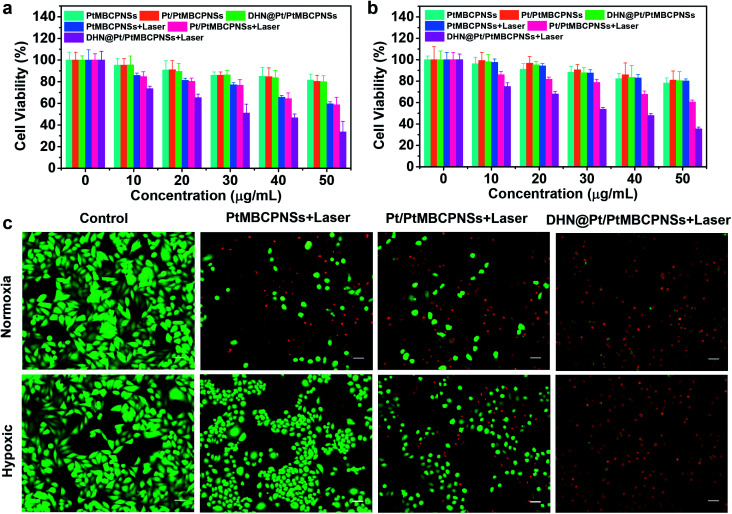
Synergistic PDT and chemotherapy *in vitro*. Cell viability levels of HeLa cells incubated with different concentrations of PtMBCPNSs, Pt/PtMBCPNSs, and DHN@Pt/PtMBCPNSs under (a) normoxic and (b) hypoxic conditions for 24 h. (c) Live/dead staining of HeLa cells with different treatments under normoxic and hypoxic conditions. (Scale bar = 40 μm.)

The cytotoxicity levels of the Pt/PtMBCPNSs and DHN@Pt/PtMBCPNSs were evaluated using HeLa cells. In the normoxic environment, the viability levels of HeLa cells incubated with PtMBCPNSs, Pt/PtMBCPNSs, and DHN@Pt/PtMBCPNSs for 24 h were higher than 80% with no light irradiation (Fig. 4a). Under laser illumination, DHN@Pt/PtMBCPNSs exhibited higher cytotoxicity than did the PtMBCPNSs and Pt/PtMBCPNSs, attributed to the excess ^1^O_2_ produced by DHN@Pt/PtMBCPNSs further oxidizing non-toxic DHN (Fig. S30a†) into toxic juglone (Fig. S30b†). This process hence was shown to achieve a synergistic chemo-photodynamic therapy of cancer. As displayed in Fig. 4b, under the hypoxic conditions, PtMBCPNSs showed weak cytotoxicity under laser irradiation. However, both Pt/PtMBCPNSs and DHN@Pt/PtMBCPNSs presented relatively high cytotoxicity levels under laser irradiation. Moreover, a similar cell viability was measured with or without laser irradiation in cells treated with DHN@PtMBCPNSs and DHN@Pt/PtMBCPNSs under normoxic conditions. However, in the hypoxic environment, DHN@Pt/PtMBCPNSs showed obvious cytotoxicity, while DHN@PtMBCPNSs showed weak cytotoxicity under the same conditions (Fig. S31†). These results suggested that Pt/PtMBCPNSs could alleviate hypoxia by producing O_2_ and hence also reduce the resistance of tumors to drugs.

Then live/dead cell staining experiments were used to evaluate the synergistic treatment effect (Fig. 4c). Here, live cells were stained with green fluorescent calcein acetoxymethyl ester (calcein AM), and dead cells were stained with red fluorescent propidium iodide (PI). In the control experiments, we only observed bright green fluorescence. After irradiation with a 660 nm-wavelength laser (100 mW cm^−2^) for 10 minutes under normoxic and hypoxic environments, the cells treated with Pt/PtMBCPNSs as well as those treated with DHN@Pt/PtMBCPNSs showed red fluorescence. However, the cells treated with PtMBCPNSs showed no red fluorescence under hypoxic conditions, indicating that PtMBCPNSs cannot change tumor hypoxia. Based on the proportion of red and green fluorescent cells, we observed the PDT and chemotherapy effect of DHN@Pt/PtMBCPNSs to be significantly better than that of Pt/PtMBCPNSs, consistent with the CCK-8 assay results (shown in Fig. 4a and b) and demonstrating that the DHN@Pt/PtMBCP nanocapsule in combination with chemo-photodynamic therapy can kill HeLa cells.

### 2.8 *In vivo* antitumor efficacy of DHN@Pt/PtMBCPNSs

The excellent synergistic chemotherapeutic and phototherapeutic effects of the DHN@Pt/PtMBCPNS system at the cellular level prompted us to further study its *in vivo* anticancer effect. Prior to *in vivo* therapy for mice bearing the Hepatoma 22 (H22) tumor model, the biodistribution and metabolism of DHN@Pt/PtMBCPNSs were investigated. Mice bearing H22 tumors were intravenously injected with DHN@Pt/PtMBCPNSs. Samples including blood, urine, feces, tumor, and major organs (heart, liver, spleen, lung, kidney) were collected at various times post injection to be analyzed using inductively coupled plasma atomic emission spectrometry (ICP-AES). As shown in Fig. S32,† 10.6% ID per g Pt/PtMBCPNSs was achieved in tumors within 24 h of intravenous injection, indicative of the passive tumor targeting capabilities of DHN@Pt/PtMBCPNSs. Through seven days of tracking experiments on the biodistribution of DHN@Pt/PtMBCPNSs in mice, it was found that the DHN@Pt/PtMBCPNSs were gradually cleared from the liver and spleen. Time-dependent ICP-AES analysis of the amount of Pt showed that large amounts of injected DHN@Pt/PtMBCPNSs were present in the feces within one week, while only small amounts of DHN@Pt/PtMBCPNSs were excreted through the urine (Fig. S33†), demonstrating that DHN@Pt/PtMBCPNSs were mainly removed from mice *via* liver and spleen pathways. Moreover, DHN@Pt/PtMBCPNSs exhibited a high blood-circulation capacity, reaching 4.3% ID per g within 24 h, a value significant for tumor targeting (Fig. S34†).

**Fig. 5:**
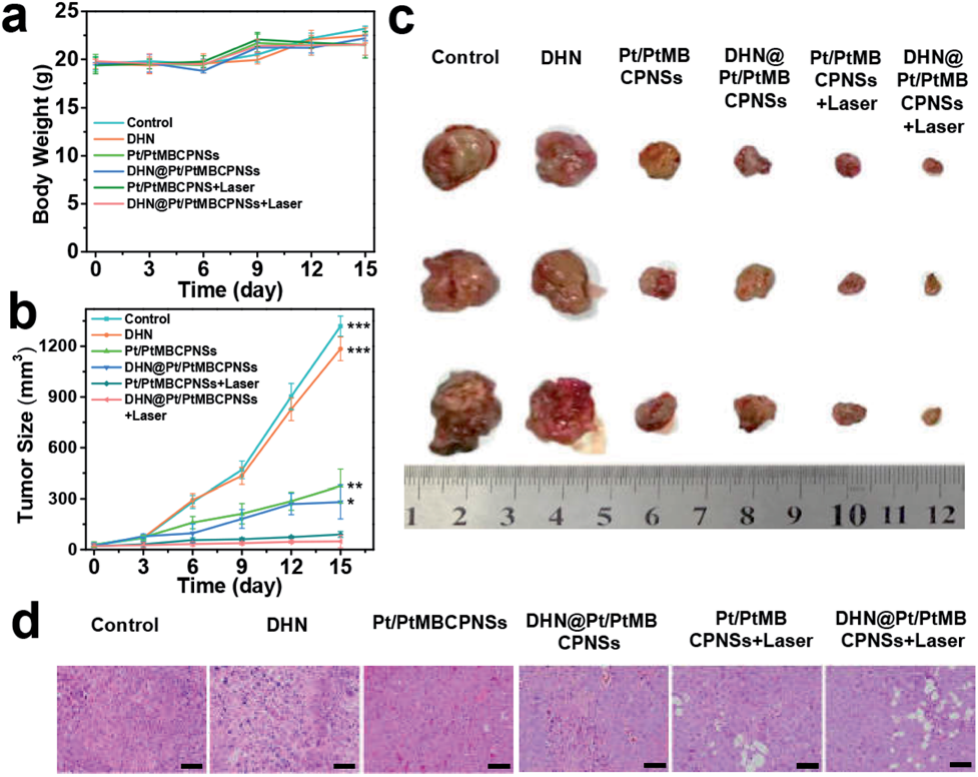
Therapeutic effects of DHN@Pt/PtMBCPNSs. (a) Body weights and (b) tumor sizes of tumor-bearing mice after various treatments. (c) Digital photographs of excised tumors after 15 days of various treatments. *P* values were calculated using the *t*-test (*P < 0.05, **P < 0.01, and ***P < 0.001) to compare other groups with DHN@Pt/PtMBCPNSs + laser. (d) H&E staining of tumor tissue samples harvested from corresponding mice after 15 days of various treatments. (Scale bar = 50 μm.)

Tumor-bearing mice with H22 cancer were divided into six groups, and then these groups were intravenously injected with various agents and corresponding treatments: saline, DHN, Pt/PtMBCPNSs, DHN@Pt/PtMBCPNSs, Pt/PtMBCPNSs + laser, and DHN@Pt/PtMBCPNSs + laser, respectively. The biocompatibility and therapeutic effects of the materials were evaluated by monitoring the body weights of mice and the volumes of the tumors every 3 days. As shown in Fig. 5a, no significant weight changes were observed during the entire treatment process, and the main organs (heart, liver, spleen, lung, kidney) of the mice were not damaged (Fig. S35a†), which further proved the high biological safety of DHN@Pt/PtMBCPNSs. Remarkably, compared with the other five groups, tumor growth in the group treated with DHN@Pt/PtMBCPNSs + laser was significantly inhibited, and the tumor inhibition rate was 95.5% after 15 days of treatment (Fig. 5b and c).

In addition, Pt/PtMBCPNSs and DHN@Pt/PtMBCPNSs were found to be able to inhibit the growth of tumors without light irradiation. This ability was attributed to the intracellular GSH reducing Pt(iv) in Pt/PtMBCPNSs to cytotoxic Pt(ii)^50–52^ (Fig. S36–S38, detailed discussion in the ESI†). Moreover, the cytokine levels in the sera of mice treated with saline, DHN@Pt/PtMBCPNSs, and DHN@Pt/PtMBCPNSs + laser were examined. As displayed in Fig. S38,† low levels of TNF-α, IL-6, and IL-1β were found in the sera of these mice. This result indicated that the DHN@Pt/PtMBCPNSs did not induce an immune response. Moreover, hematoxylin and eosin (H&E) staining results (Fig. 5d) showed that mice treated with DHN@Pt/PtMBCPNSs + laser exhibited significant cancer cell necrosis in tumor sections. These experimental results provided strong evidence for an excellent anticancer effect of DHN@Pt/PtMBCPNSs under laser irradiation. H&E staining showed no significant physiological changes in the main organs including the heart, liver, spleen, lungs, and kidneys as a result of the DHN@Pt/PtMBCPNS treatment (Fig. S35b†). That is, the DHN@Pt/PtMBCPNSs showed no significant systemic toxicity during the therapy.

## 3. Conclusions

In conclusion, for the first time, a spatiotemporally controlled O_2_ and ^1^O_2_ self-sufficient Pt/PtMBCPNS nanophotosensitizer was successfully developed for the *in vivo* high-yield synthesis of drugs and synergistic chemo-photodynamic therapy for hypoxic cancer. After being endocytosed, the photosensitizer with its good catalase-like activity and remarkably enhanced photosensitization mediated a “H_2_O_2_ → O_2_ → ^1^O_2_” cascade reaction, thus improving the hypoxic environment of the tumor and enhancing the PDT. Simultaneously, the excess ^1^O_2_ photooxidized the prodrug DHN to result in the *in situ* high-yield generation of the anticancer drug juglone, which induced localized cytotoxicity and minimized the toxic side effects of cytotoxic drugs. Importantly, by spatiotemporally adjusting the level of generated O_2_ in the TME, the nanophotosensitizer could ensure an obvious antitumor effect from the produced drugs and reduce drug resistance. In both cellular and *in vivo* experiments, the nanophotosensitizer induced significant cytotoxicity in cancer cells and resulted in the remarkable and specific inhibition of tumor growth. We believe that such a tumor-selective nanophotosensitizer, with the spatiotemporally controlled *in situ* high-yield synthesis of drugs and generation of O_2_ at the tumor site, represents a robust nanomedicine strategy to overcome the current limitations of the low efficiency of *in situ* drug synthesis and the drug resistance of hypoxic tumors and achieve ultimately clinical applications.

## 4. Experimental

### 4.1 Synthesis of Pt/PtMBCPNSs

A volume of 1 mL of an aqueous solution of 1.0 mM MB, a mass of 10 mg of PVP, and a volume of 10 mL of an aqueous solution of 0.4 mM H_2_PtCl_6_ were mixed in a reaction vessel. The reaction mixture was irradiated with visible light (420 nm < *λ* < 780 nm filter) for 30 min at a constant temperature of 37 °C. The Pt/PtMBCPNS product was gathered by performing centrifugation (10 000 rpm), and washing with ethanol several times until the supernatant was colorless.

### 4.2 Loading DHN into Pt/PtMBCPNSs (DHN@Pt/PtMBCPNSs)

The loading of prodrug molecules and calculation of their loading content were based on previously published methods with some modifications.^13,49^ Pt/PtMBCPNSs (2.0 mg) were dispersed in 2 mL of a DHN/ethanol solution (2 mg mL^−1^) and then magnetically stirred at room temperature for 24 h. The DHN@Pt/PtMBCPNSs were separated from the dispersion by performing centrifugation, and washed with ethanol three times. The supernatant was collected to measure, using UV-Vis absorption spectroscopy, the loading content of the prodrug.

### 4.3 Extracellular detection of O_2_ generation

Foe each of three experiments, a volume of 40 mL of a PBS buffer solution (pH 6.0) containing 400 μL of H_2_O_2_ (10 mM) and a volume of 100 μL of 10 mg mL^−1^ of one of three different samples (Pt/PtMBCPNSs, PtMBCPNSs, or H_2_O) were added into a 50 mL round-bottom flask, and the resulting solution was bubbled with N_2_ for 15 minutes to remove dissolved O_2_. Then, real-time generation of O_2_ was monitored at 37 °C by using a JPBJ-609L meter for measuring the amount of dissolved oxygen. Also, a PBS buffer solution (pH 6.0) by itself and Pt/PtMBCPNSs were tested for O_2_ production under the same conditions. At the same time, successive generation of O_2_ resulting from adding 400 μL of H_2_O_2_ (10 mM) to a suspension of Pt/PtMBCPNSs every 30 minutes was also tested by using the JPBJ-609L meter.

### 4.4 Extracellular detection of ^1^O_2_ generation

DPBF was chosen as a probe to detect the ^1^O_2_ generated by Pt/PtMBCPNSs, MB or PtMBCPNSs with or without H_2_O_2_ under 660 nm-wavelength laser irradiation, because DPBF is readily oxidized by active oxygen species and as a result undergoes structural changes that cause a gradual decrease of its absorption at 410 nm. Typically, a volume of 100 μL of 10 mg mL^−1^ Pt/PtMBCPNSs, MB or PtMBCPNSs was added to the PBS buffer solution (pH 6.0) containing DPBF (0.28 mg mL^−1^). Changes in the concentration of DPBF in the solution was monitored by acquiring corresponding UV-Vis absorption spectra with or without H_2_O_2_ under laser irradiation (660 nm, 100 mW cm^−2^).

### 4.5 Electron paramagnetic resonance (EPR) trapping measurements

Briefly, a volume of 30 μL of a Pt/PtMBCPNS- or MB-dispersed aqueous solution (10 mg mL^−1^) was added to 1 mL of PBS buffer solution (pH 6.0) containing 10 μL of TEMP (5 M) to identify the ^1^O_2_. Before EPR measurement, the above solution was irradiated with a 660 nm-wavelength laser for three minutes.

### 4.6 Photooxidation of DHN by Pt/PtMBCPNSs

A volume of 100 μL of Pt/PtMBCPNSs (10 mg mL^−1^) was added to 30 mL of a DMSO/PBS buffer solution (pH 6.0) (0.1/100, v/v) containing DHN (1.0 × 10^−4^ M) in a 50 mL round-bottom flask. After the reaction solution was irradiated with a laser (660 nm, 100 mW cm^−2^), the concentration changes of the reactant and product in the solution were tracked by acquiring and inspecting their UV-Vis absorption spectra at various time points.

### 4.7 Intracellular ROS detection

DCFH-DA was used as an indicator to measure the levels of intracellular ROS under normoxic or hypoxic conditions. In laser confocal Petri dishes, samples of HeLa cells were treated with, respectively, PtMBCPNSs, Pt/PtMBCPNSs, and DHN@Pt/PtMBCPNSs (40 μg mL^−1^) for 2 h. The original medium was replaced with a 1 × 10^−6^ M DCFH-DA staining solution. After another 30 minutes of incubation, the cells were irradiated with a laser (660 nm, 100 mW cm^−2^) for 10 min. For the control group, the cells treated with Pt/PtMBCPNSs and mixed with DCFH-DA were cultured in the dark. All of the cells were washed, and then imaged by performing CLSM. The contents of ROS in the various cells treated using different methods were read with the help of the CLSM software.

### 4.8 *In vivo* combination therapy

The tumor-bearing mice of the various mouse groups (*n* = 3) were intravenously injected and treated with saline, DHN (2 mg kg^−1^), Pt/PtMBCPNSs (10 mg kg^−1^), DHN@Pt/PtMBCPNSs (12 mg kg^−1^), Pt/PtMBCPNSs (10 mg kg^−1^), and DHN@Pt/PtMBCPNSs (12 mg kg^−1^), respectively. After 24 h, the mice of the Pt/PtMBCPNS and DHN@Pt/PtMBCPNS groups were irradiated with a 660 nm-wavelength laser (200 mW cm^−2^) for 10 minutes. The administration was repeated every 2 days. The tumor volume was monitored every 3 days over the course of 15 days with a digital caliper to evaluate the tumor inhibitory effects of various treatments.

## Ethical statement

All animal experiments were performed according to the Guidelines for Safe Work Practices approved by the Committee on Ethics of Human Specimens and Animal Experiments at Lanzhou University Second Hospital.

## Conflicts of interest

There are no conflicts to declare.

## Supplementary Material

SC-011-D0SC02387F-s001
