# Supplementary material for: Spatiotemporally controlled O2 and singlet oxygen self-sufficient nanophotosensitizers enable the in vivo high-yield synthesis of drugs and efficient hypoxic tumor therapy
Source: Chem Sci. 2020 Jul 28;11(33):8817–27. doi: 10.1039/d0sc02387f (PMC8163376; doi:10.1039/d0sc02387f)
Supplement: SC-011-D0SC02387F-s001 [file SC-011-D0SC02387F-s001.pdf]

Electronic Supplementary Material (ESI) for Chemical Science.
This journal is © The Royal Society of Chemistry 2020

Supporting Information

Spatiotemporally controlled O_2 and singlet oxygen self-sufficient nanophotosensitizers enable *in vivo* high-yield synthesis of drugs and efficient hypoxic tumor therapy

Suisui He[a], Siyu Lu[c], Sha Liu[a], Tianrong Li[a], Jieling Li[a], Shihao Sun[a], Meilin Liu[a], Kun Liang[a], Xu Fu[b], Fengjuan Chen[a], GenPing Meng[a], Lang Zhang[d], Jun Hai*[,a], Baodui Wang*[,a]

[a]*State Key Laboratory of Applied Organic Chemistry and Key Laboratory of Nonferrous Metal Chemistry and Resources Utilization of Gansu Province, Lanzhou University, Gansu, Lanzhou, 730000. China. E-mail:* haij@lzu.edu.cn; wangbd@lzu.edu.cn.

[b]*Laboratory of Emergency Medicine, Lanzhou University Second Hospital, Lanzhou, 730000, China.*

[c]*College of Chemistry and Molecular Engineering, Zhengzhou University, Zhengzhou, 450001, China.*

[d]*Institute of Anatomy and Histology & Embryology, School of Basic Medical Sciences, Lanzhou University, Lanzhou, China, 730000, China.*

Experimental Section

Materials

All commercially available compounds were used without further purification unless otherwise noted. Chloroplatinic acid hexahydrate ($H_2PtCl_6·6H_2O$, 99+%, Pt ≥ 38%) was purchased from Innochem. Polyvinyl pyrrolidone (PVP) was purchased from Sigma-Alorich. Methylene blue (for biological staining, dye content ≥ 90%) and tris (4,7-diphenyl-1,10-phenanthroline) ruthenium(II) dichloride complex $[Ru(dpp)_3]Cl_2$ (RDPP, ≥ 95%) were purchased from Aladdin reagents. Hydrogen peroxide (H_2O_2) was purchased from Sinopharm Chemical Reagent Co. Ltd. L-glutathione reduced (GSH, > 98%) was purchased by Solarbio. 1,3-diphenylisobenzofuran (DPBF, 97%), 2,2,6,6-tetramethyl-4-piperidone (TEMP, 98%), 2′,7′-dichlorofluorescein diacetate (DCFH-DA, > 97%), 1,5-dihydroxynaphthalene (1,5-DHN, 98%), 1-5-hydroxy-1,4-naphthalenedione (naphthol, 99%), 1,6-dihydroxynaphthalene (1,6-DHN, 99%), and juglone (97%) were purchased from Energy Chemical. Calcein-AM/PI double stain kit was purchased from Beyotime. The Mouse TNF-α (Tumor Necrosis Factor Alpha) ELISA Kit, Mouse IL-6 (Interleukin 6) ELISA Kit, and Mouse IL-1β (Interleukin 1 Beta) ELISA Kit were purchased from Elabscience Biotechnology Co., Ltd.

Measurements

The morphologies of the samples were observed by a field emission scanning electron microscope (FE-SEM, FEI, Sirion 200) and transmission electron microscope (TEM, FEI, Talos 200s). X-ray powder diffraction (XRD) patterns of the nanomaterials were recorded on a Bruker AXS D8-Advance diffractometer with Cu Kα radiation (λ = 1.5418 Å). Fourier transform infrared spectra (FT-IR) were obtained on a Nicolet FT-170SX spectrometer. UV−vis absorbance measurements were recorded on Shimadzu UV-1750. X-ray photoelectron spectroscopy (XPS) measurements were performed on a PHI-5702 multifunctional spectrometer using Al Kα radiation. The XPS profiles were fitted by the Gaussian-Lorentzian method after background subtraction using Shirley's method. ^1H NMR spectra were recorded on a JNM-ECS400 spectrometer. Oxygen production was measured with an oxygen probe (JPBJ-609L Portable Dissolved Oxygen Meter). Photosynthesis was performed using a xenon lamp (HSX-F/UV 300), equipped with 400−780 nm filters. An optical fiber-coupled 660 nm laser (MIL-N-660-5W) was purchased from Changchun New Industries Optoelectronics Tech Co. Ltd. (CNI). Pt content was determined by inductively coupled plasma atomic emission spectrometry (ICP-AES, Varian VISTA-MPX).

Synthesis of PtMBCPNSs

1 mL of 1.0 mM MB aqueous solution, 10 mg of PVP, and 10 mL of 0.4 mM H_2PtCl_6 aqueous solution were added to the reaction vessel, respectively. The reaction mixture was irradiated by visible light (420 nm < λ < 780 nm filter) for 1 min in a constant temperature water bath at

37 ^{o}C. The product of Pt/PtMBCPNSs was collected by centrifugation (10000 rpm) and washed with ethanol for several times until the supernatant was colorless.

Statistical method of size distribution

A statistical software (Nano Measurer 1.2) was used to randomly count 100 nanoparticles. The exported data was then analyzed and plotted in a professional function drawing software (Origin 9) to obtain the size distributions of nanoparticles.

Calculation of photooxidation yield

The consumption of DHN was monitored by the reduced intensity of the absorption peak at 298 nm, and the concentration of DHN was calculated based on its molar extinction coefficient (ε = 7664 M^{-1} cm^{-1}).[1] The generation of juglone was monitored by the increased intensity of the absorption peak at 419 nm. The molar extinction coefficient (ε = 3500 M^{-1} cm^{-1})[1] was used to calculate the concentration of juglone, and the yield of juglone was obtained by calculating the final amount of juglone and the initial amount of DHN.[1] The structure of the target product was further proved by ^{1}H NMR.

The GSH-triggered DHN release

DHN@Pt/PtMBCPNSs (2 mg) were packaged into a dialysis bag (MWCO = 3500), and then immersed within 15 mL PBS or aqueous solution containing GSH (10 mM) at 37 °C in a beaker. At different time points, 2.0 mL solution was collected to determine the concentration of DHN using UV−vis spectra, and then 2.0 mL fresh PBS or aqueous solution containing GSH was added to the beaker to make up for the reduced solution volume. Three independent experiments were carried out to minimize the deviations.

Cell culture

Human cervical carcinoma (HeLa) cells were purchased from the cell bank of the Chinese Academy of Sciences (Shanghai, China). HeLa cells were cultured in DMEM with an atmosphere of 21% O_2 and 5% CO_2 at 37 ^{o}C to mimic the normoxic environment. Hypoxic condition was achieved by incubating the cells under a hypoxic incubator with an atmosphere of 1% O_2, 5% CO_2, and 94% N_2. Cell viability was detected by a cell counting kit-8 (CCK-8, Beyotime Biotechnology) test following the manufacturer's instructions.

Cell uptake of DHN@Pt/PtMBCPNSs

HeLa cells were inoculated into 12-well plates at a density of 1 × 10^5 cells per well. After incubating for 24 h, 40 µg of DHN@Pt/PtMBCPNSs were added and the cells were incubated for another 30 min, 1 h, and 2 h. Then the cells were washed three times with PBS to remove free nanomaterials. Finally, the cell uptake of nanomaterials was tracked by a confocal imaging system.

Intracellular O_2 detection

RDPP was used as a probe to detect intracellular O_2 levels. HeLa cells were seeded in confocal dishes for 24 h of incubation in hypoxic microenvironment. After 4 h of treatment with 5 μmol L^{-1} RDPP for 4 h at 37 ^{o}C, the cells were incubated with 40 μg mL^{-1} PtMBCPNSs, Pt/PtMBCPNSs, and DHN@Pt/PtMBCPNSs for 4 h. After washing with PBS for three times, the fluorescence images of cells were obtained at 455 nm excitation using CLSM.

Cytotoxicity assay

The cytotoxicity against HeLa cells was measured by CCK-8 assay. The cells were cultured in 96-well plates and incubated for 24 h at 37 °C under normoxic or hypoxic condition. Thereafter, DHN, PtMBCPNSs, Pt/PtMBCPNSs, DHN@PtMBCPNSs, DHN@Pt/PtMBCPNSs, and juglone with gradient concentrations were added into each well. After incubating for another 2 h, the DHN, PtMBCPNSs, Pt/PtMBCPNSs, DHN@PtMBCPNSs, and DHN@Pt/PtMBCPNSs groups were irradiated with a 660 nm laser (100 mW cm^{-2}) for 10 min. The concentration of PtMBCPNSs and Pt/PtMBCPNSs were 0, 10, 20, 30, 40, and 50 μg·mL^{-1}, and the concertation of DHN@PtMBCPNSs and DHN@Pt/PtMBCPNSs were 0, 12, 24, 36, 48, and 60 μg·mL^{-1}. Moreover, the concertation of DHN was 0, 2, 4, 6, 8, 10, and 12 μg·mL^{-1}, which was calculated based on the loading content of DHN in DHN@Pt/PtMBCPNSs, and the concentration of juglone was 0, 2, 4, 8, 12, 16, and 20 μg·mL^{-1}. As the control groups, the same experiments were conducted in the dark without illumination. Afterward, the cells were cultured for 24 h, 48 h, and 72 h, and then 10 μL of CCK-8 per well was added and incubated for 2 h at 37 °C under 5% CO_2. The optical density was measured at 450 nm using a microplate reader (Wallac 1420 Victor).

Cell live/dead assay

HeLa cells were seeded onto CLSM exclusive culture disks (φ = 15 mm) and then were incubation with saline, PtMBCPNSs, Pt/PtMBCPNSs, DHN@PtMBCPNSs, and DHN@Pt/PtMBCPNSs for 24 h under normoxic or hypoxic condition. After removing the culture medium and rinsing the disks, a calcein solution (50 μL, 20 mM) and a PI solution (50 μL, 20 mM) were used to stain the live cells green (λ_{ex} = 490 nm, λ_{em} = 515 nm) and the dead cells red (λ_{ex} = 535 nm, λ_{em} = 617 nm). After 15 min of incubation, the cells were rinsed twice with PBS and imaged by CLSM.

Animals

Balb/c mice were obtained from Nanjing Peng Sheng Biological Technology Co. Ltd. All animal experiments were performed according to the Guidelines for Safe Work Practices approved by the Committee on Ethics of Human Specimens and Animal Experiments at Lanzhou University Second Hospital. Hepatoma 22 (H22) cancer-bearing mouse models were

generated by subcutaneous injection 1×10^5 cells in 50 μL PBS onto the back of each mouse.

***In vivo* biodistribution and metabolize evaluation**

To study the biodistribution and metabolize *in vivo* of DHN@Pt/PtMBCPNSs, mice were intravenously injected with DHN@Pt/PtMBCPNSs. Samples including blood, urine, and feces were collected at different time post injection. And mice were sacrificed at different time points. Tumors and major organs (heart, liver, spleen, lung, and kidney) were harvested. Typically, blood, urine, feces, tumors and major organs were dissolved in chloroazotic acid. The mixtures were heated and the Pt amounts in the above samples were assessed via ICP-AES measurement.

H&E staining

For evaluating the pathological damages to tumors of different treatments, the tumor tissues in each group were retrieved after the 15 days treatment and processed for H&E staining analysis.

ELISA analysis

Mice were treated with saline, DHN@Pt/PtMBCPNSs, and DHN@Pt/PtMBCPNSs + Laser. Serum samples of mice were collected 24 h post-irradiation for pro-inflammatory cytokines (TNF-α, IL-6, and IL-1β) analysis by ELISA.

Statistical analysis

The data were presented as means ± standard deviation (SD). The statistical significance between two groups was obtained through two-tailed student *t*-test (*p < 0.05, **p < 0.01, and ***p < 0.001).

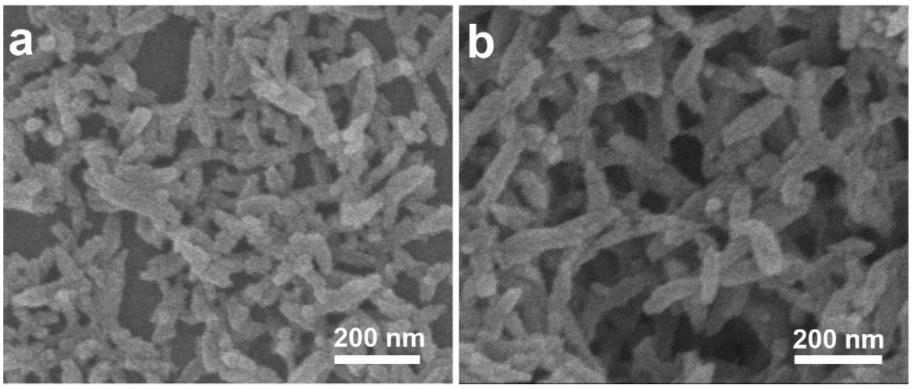

Figure S1. SEM images of (a) PtMBCPNSs and (b) Pt/PtMBCPNSs.

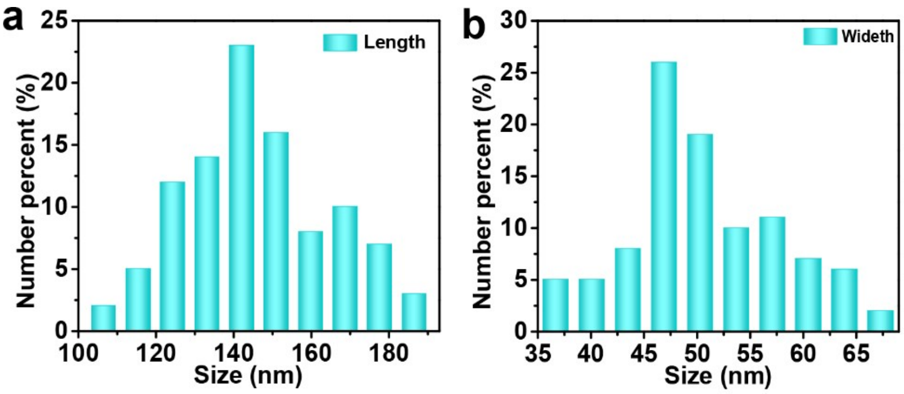

Figure S2. The size distribution of (a) the length and (b) the width of Pt/PtMBCPNSs.

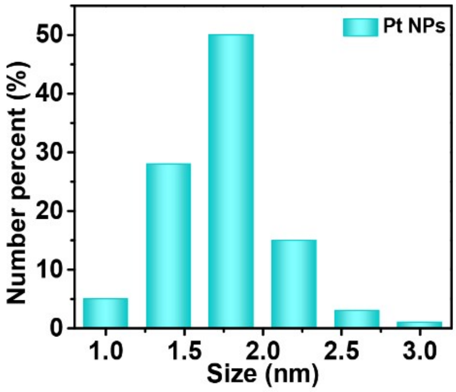

Figure S3. The size distribution of Pt NPs on Pt/PtMBCPNSs.

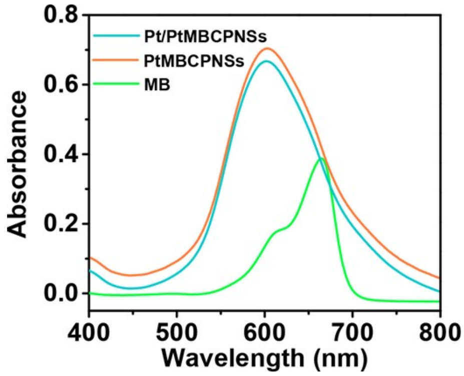

Figure S4. UV−vis absorption spectra of MB, PtMBCPNSs, and Pt/PtMBCPNSs.

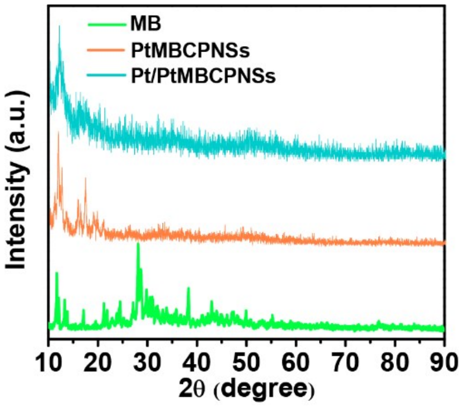

Figure S5. Powder XRD pattern of MB, PtMBCPNSs, and Pt/PtMBCPNSs.

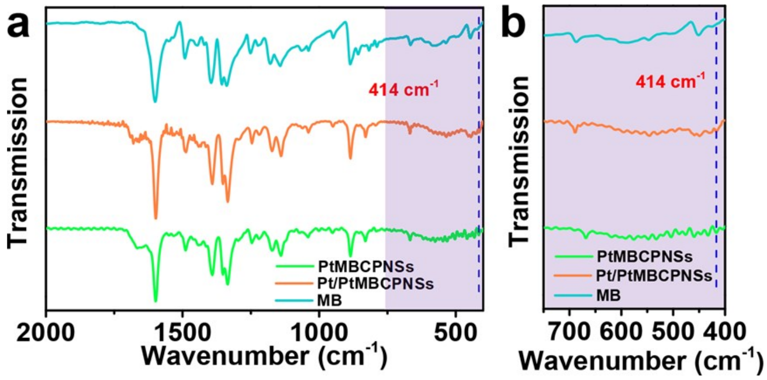

Figure S6. (a) FT-IR spectra of MB, PtMBCPNSs, and Pt/PtMBCPNSs. (b) Magnified view of the purple area in **Figure S6a**.

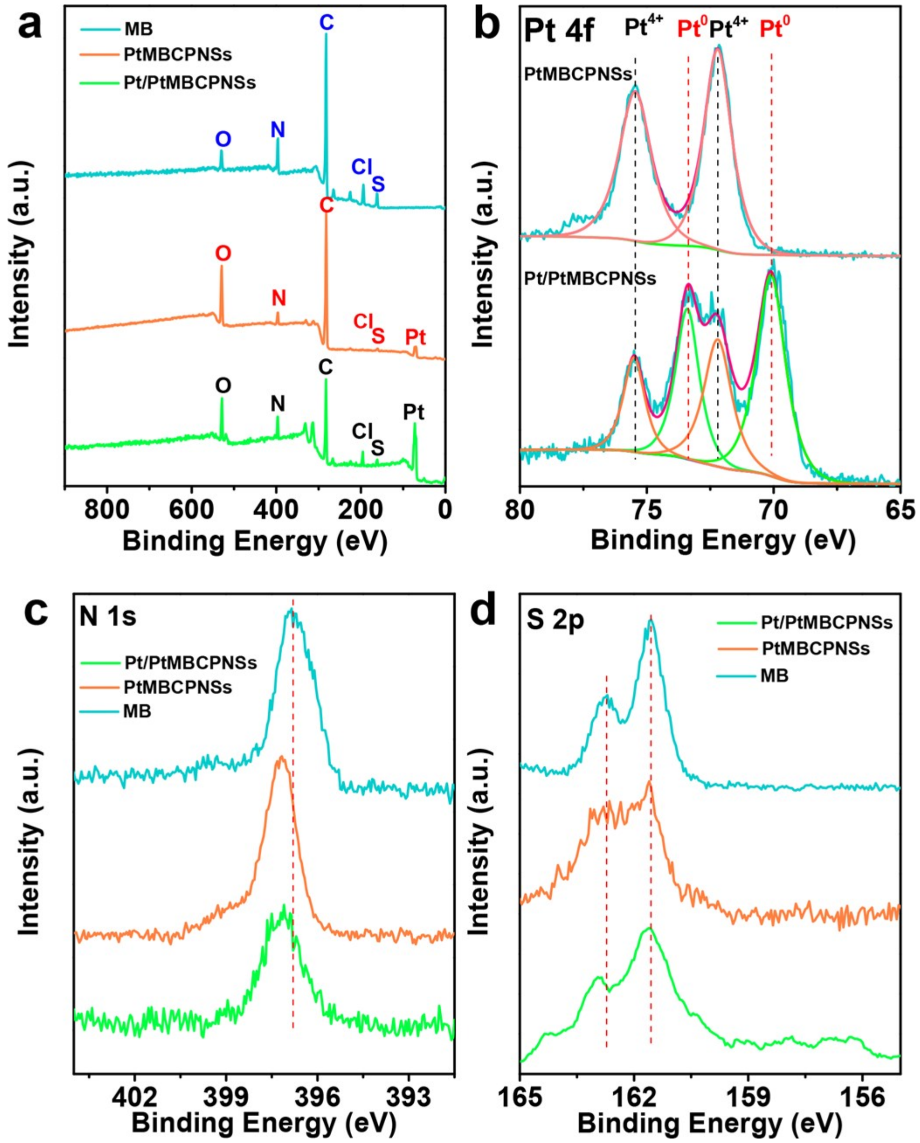

Figure S7. (a) Survey XPS spectra and high resolution (b) Pt 4f, (c) N 1s, and (d) S 2p XPS spectra of MB, PtMBCPNSs, and Pt/PtMBCPNSs.

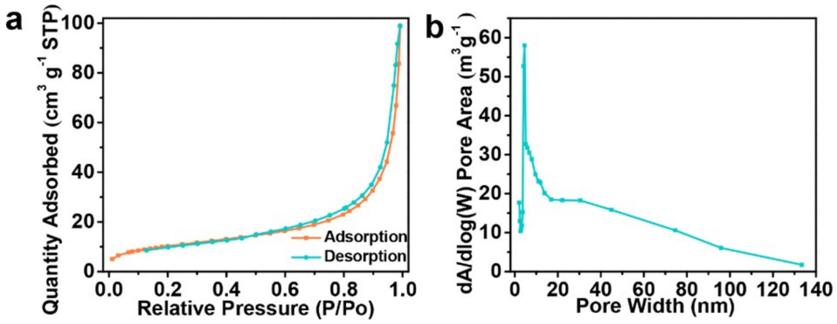

Figure S8. (a) N_2 adsorption-desorption isotherm and (b) pore size distribution of Pt/PtMBCPNSs.

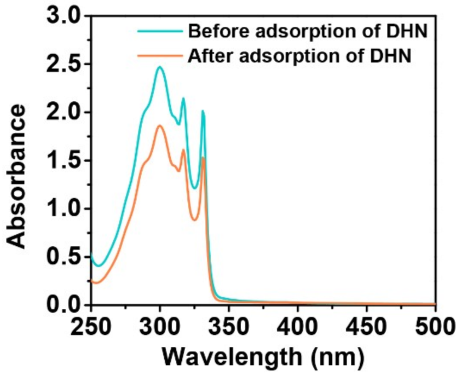

Figure S9. UV−vis absorption spectra of DHN (a) before and (b) after adsorption.

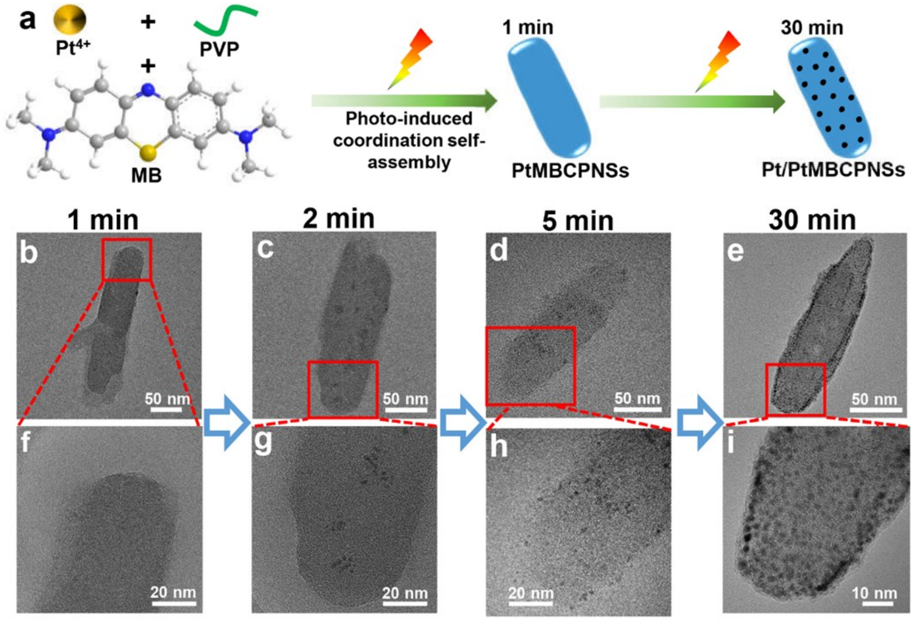

Figure S10. (a) Schematic of the Pt/PtMBCPNSs synthetic process. (b−i) TEM images of shuttle-shaped Pt coordination polymer at different times: (b, f) 1 min, (c, g) 2 min, (d, h) 5 min and (e, i) 30 min.

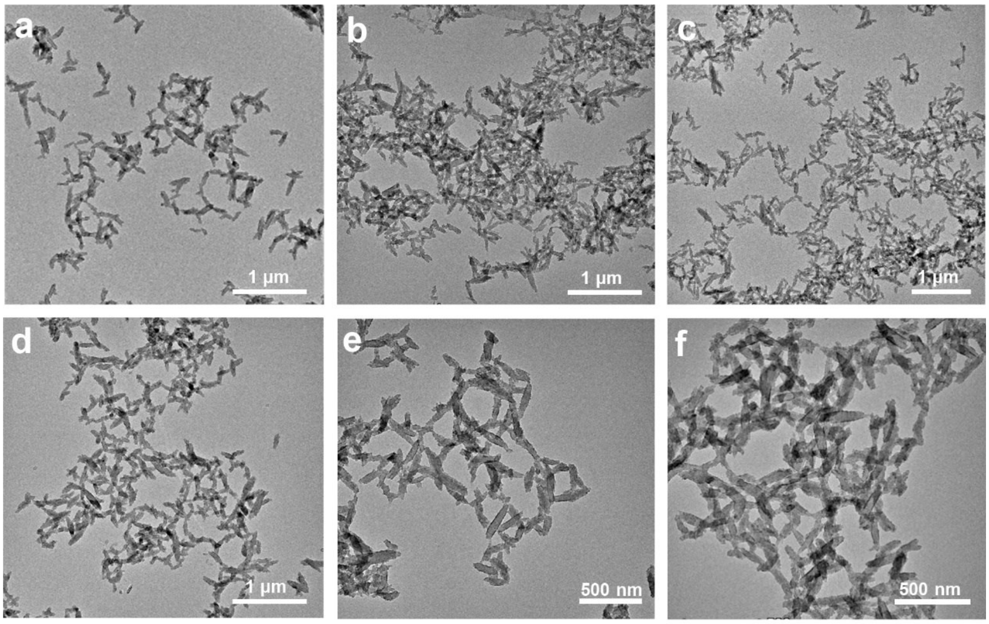

Figure S11. TEM images of Pt/PtMBCPNSs were treated under different conditions for 24 h: (a) PBS buffer solution (pH 7.4), (b) PBS buffer solution (pH 6.0), (c) PBS buffer solution (pH 5.5), (d) DMEM, (e) serum, and (f) cell lysates at physiological temperatures (37 °C).

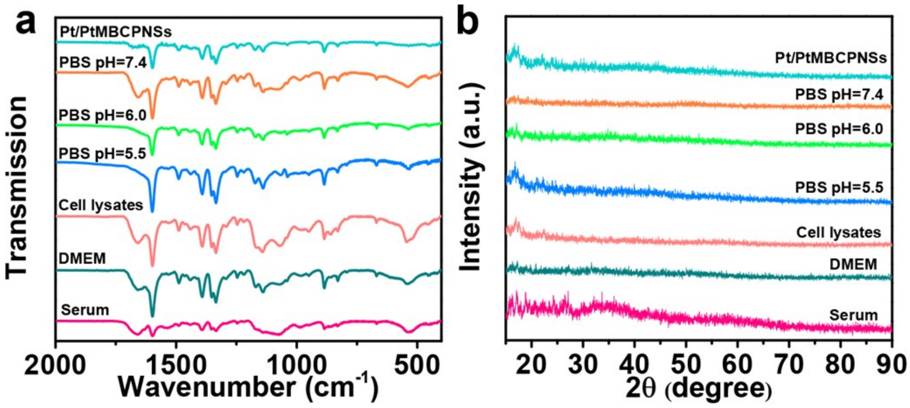

Figure S12. (a) FT-IR spectra and (b) XRD pattern of Pt/PtMBCPNSs were treated under different conditions for 24 h: PBS buffer solution (pH 7.4, 6.0, 5.5), DMEM, serum, and cell lysates at physiological temperatures (37 °C).

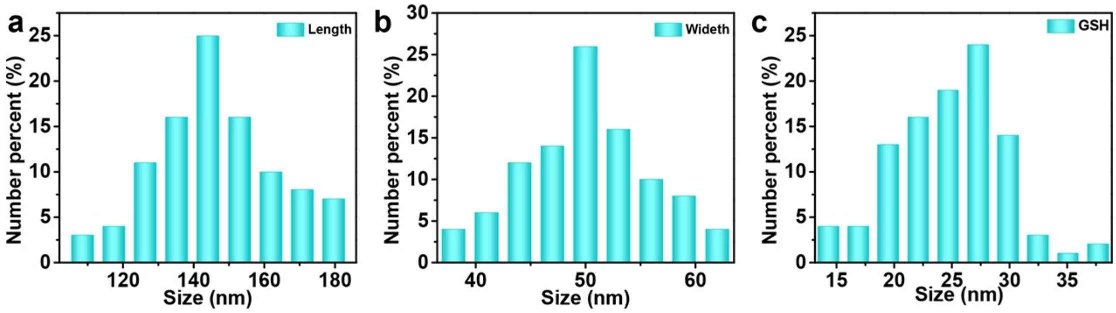

Figure S13. Size distribution of Pt/PtMBCPNSs were incubated 24 h in PBS buffer solution at (a, b) pH 7.4 or (c) pH 6.0 + 10 mM GSH.

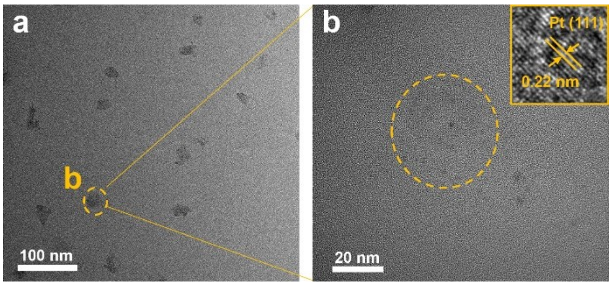

Figure S14. (a, b) TEM images at different magnifications of Pt/PtMBCPNPs and the insets of (b) shows the HRTEM image.

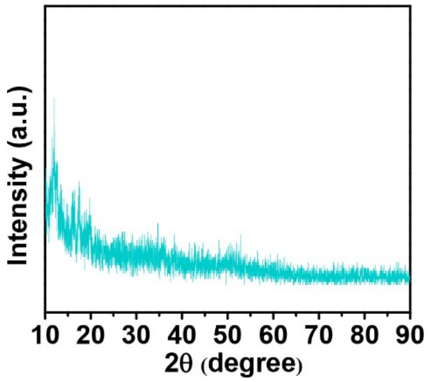

Figure S15. XRD pattern of Pt/PtMBCPNPs.

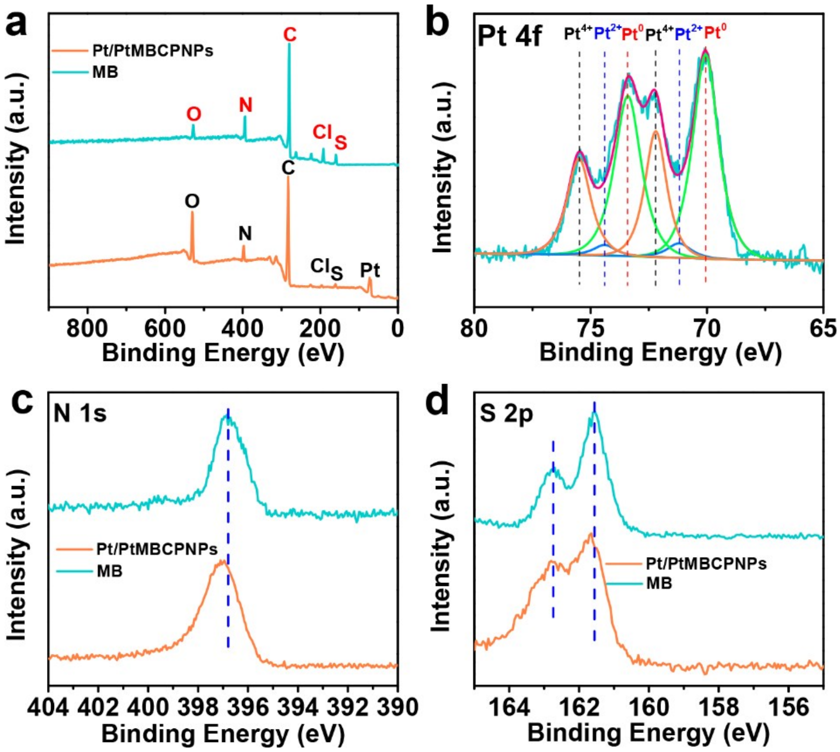

Figure S16. (a) Survey XPS pattern and high resolution (b) Pt 4f, (c) N 1s, and (d) S 2p of MB and Pt/PtMBCPNPs.

To verify the degradation product, the Pt/PtMBCPNSs were immersed in PBS buffer solution (pH 6.0) containing 10 mM GSH for 24 h, and then the degradation product was collected to confirm species and its catalytic performance. As shown in

Figure 2a–c, Pt/PtMBCPNSs could undergo remarkable transformation from the original shuttle-like shape to the final spherical shape in the presence of GSH (Figure S14a). The TEM image showed that Pt nanoparticles are uniformly distributed in the spherical Pt/PtMBCP nanoparticles (Pt/PtMBCPNPs) (Figure S14b). As observed from the HRTEM image (the inset in Figure S14b), the lattice spacing of the nanocrystal was 0.22 nm, which matches the spacing of Pt (111) crystal planes.[2] Furthermore, XRD patterns (Figure S15) showed that the final Pt/PtMBCPNPs have diffraction peaks similar to Pt/PtMBCPNSs. The above results indicated that the species of degradation product is small size Pt/PtMBCPNPs. The formed small size Pt/PtMBCPNPs were further confirmed by XPS. As shown in Figure S16a, the Pt/PtMBCPNPs also existed the Pt 4f peak, which is consistent with Pt/PtMBCPNSs. In addition, for the high resolution of Pt 4f (Figure S16b), Pt/PtMBCPNPs showed peaks belonging to Pt^{4+} species[3] at 72.2 eV and 75.5 eV as well as peaks belonging to Pt^{0} species[4] at 70.1 eV and 73.4 eV. Compared with Pt/PtMBCPNSs, peaks belonging to Pt^{2+} species[4] at 71.2 and 74.7 eV were only observed in Pt/PtMBCPNPs, indicating that a small amount of Pt^{4+} was reduced to Pt^{2+} by GSH. N 1s XPS spectrum in Figure S16c showed that the N 1s moves from 396.9 eV for MB to 397.2 eV for Pt/PtMBCPNPs, indicating that N atom coordinates with Pt^{4+}.[5] Similarly, the S 2p XPS spectrum in Figure S16d showed that S 2p moves from 161.4 and 162.9 eV for MB to 161.5 and 163.0 eV for Pt/PtMBCPNPs, revealing the coordination of S atom with Pt^{4+}.[6] Together, these results ultimately showed the degradation product is small size Pt/PtMBCPNPs.

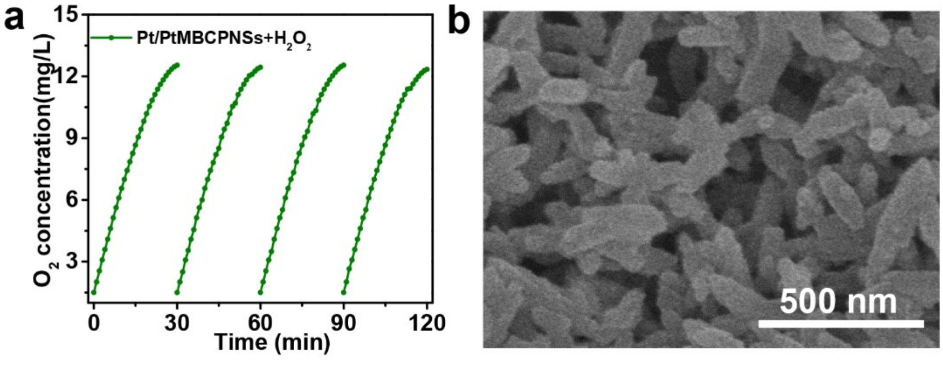

Figure S17. (a) O_2 generation by addition of H_2O_2 to the Pt/PtMBCPNSs solution. (b) SEM image of Pt/PtMBCPNSs after the four cycles tests.

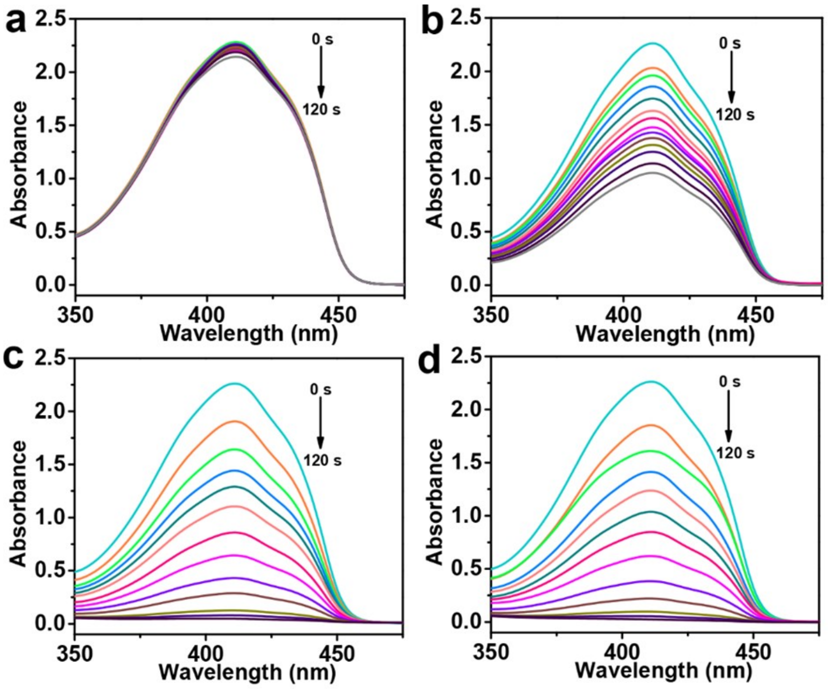

Figure S18. (a) UV−vis absorption spectra of DPBF after irradiation for different times with 660 nm laser at 100 mW cm^{-2}. (b−d) Absorption spectrum changes of DPBF under 660 nm laser irradiation with MB, PtMBCPNSs, and Pt/PtMBCPNSs.

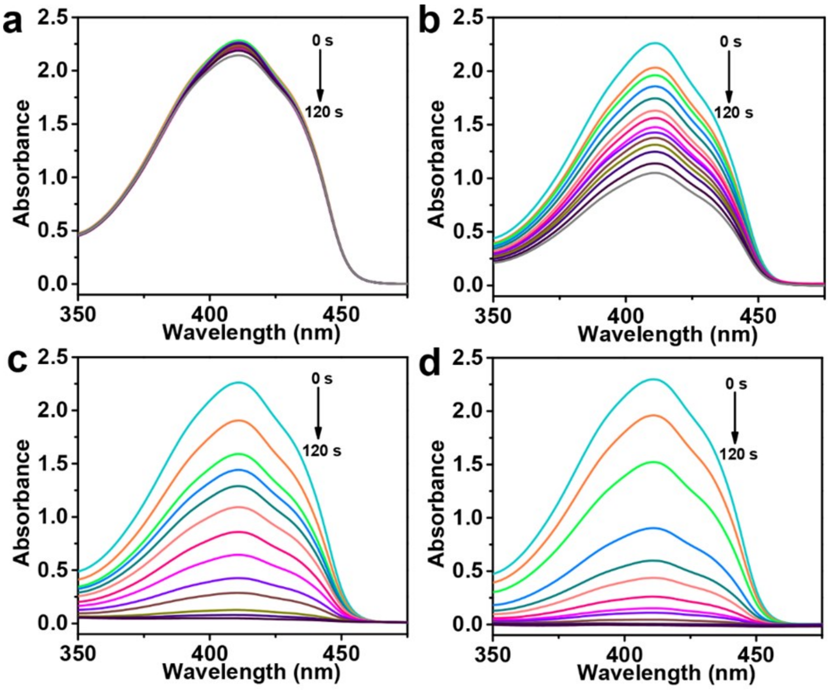

Figure S19. (a) UV−vis absorption spectra of DPBF after irradiation for different times with 660 nm laser at100 mW·cm^{-2}. (b−d) Absorption spectrum changes of DPBF under 660 nm laser irradiation with MB, PtMBCPNSs and Pt/PtMBCPNSs in the presence of H_2O_2.

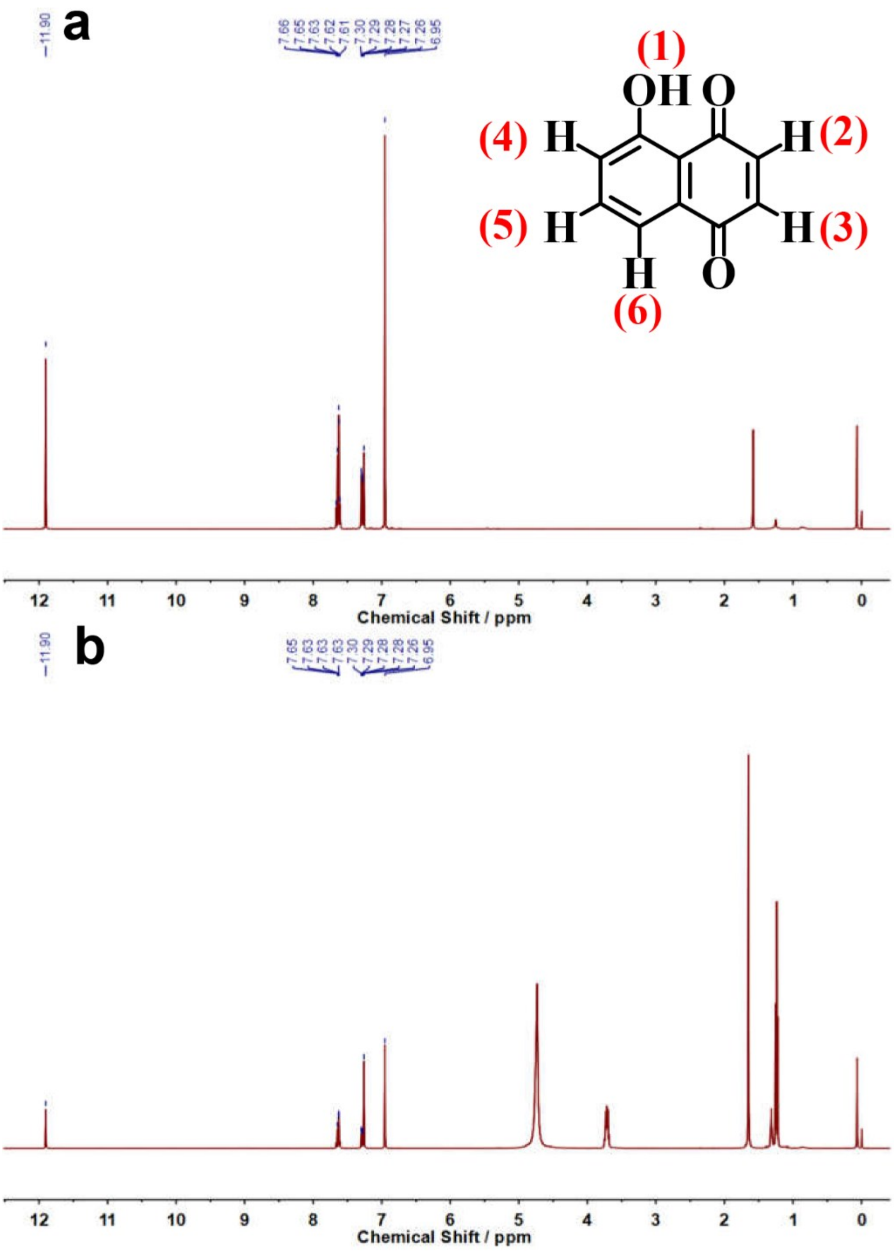

Figure S20. (a) ^{1}H NMR of commercial juglone in $CDCl_3$, (400 MHz), 25 °C. (b) ^{1}H NMR of prepared juglone in $CDCl_3$(400 MHz), 25 °C. δ (ppm)=6.95 (2H, 2# and 3#), 7.26, 7.27, 7.28, 7.29,7.30 (1H, 4#), 7.61, 7.62, 7.63 (1H, 5#), 7.65, 7.66 (1H, 6#), 11.90 (1H, 1#).

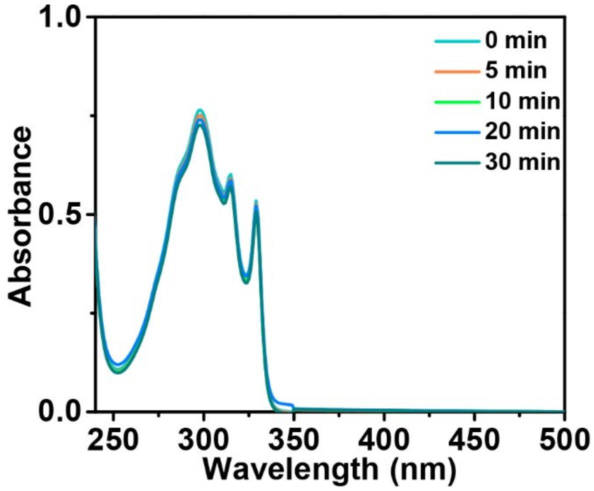

Figure S21. UV−vis absorption spectral changes for photooxidation of DHN in PBS without Pt/PtMBCPNSs.

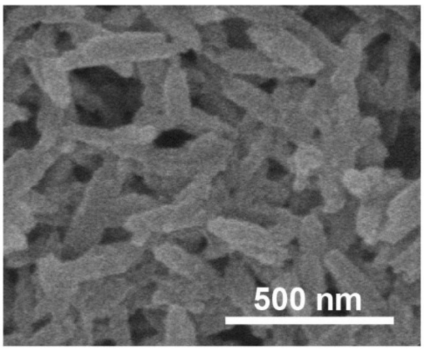

Figure S22. SEM image of Pt/PtMBCPNSs after the four cycles tests.

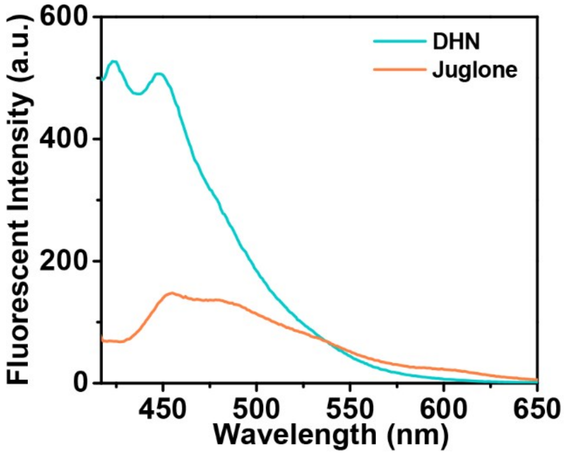

Figure S23. Fluorescence spectra of DHN and juglone (λ_{ex} = 400 nm).

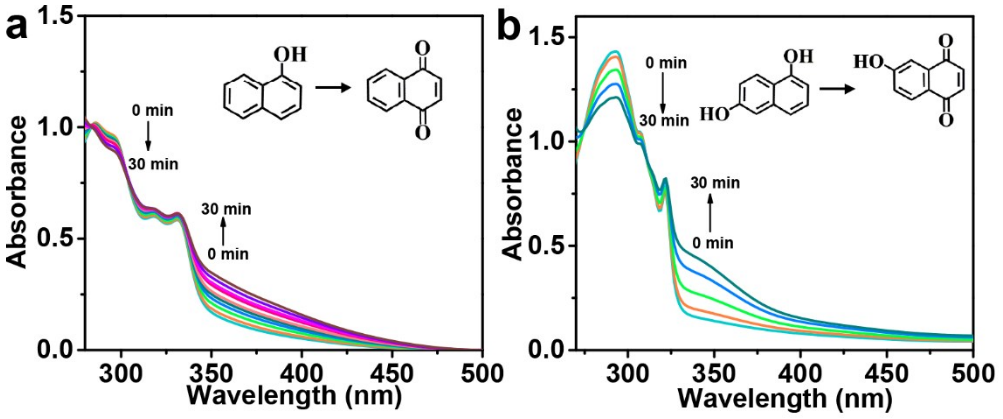

Figure S24. UV−vis absorption spectral changes for photooxidation of (a) 1-naphthol and (b) 1,6-DHN.

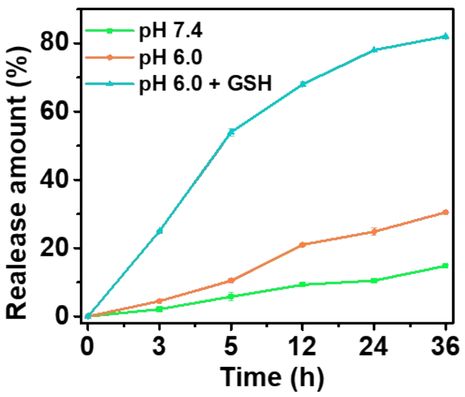

Figure S25. *In vitro* release profiles of DHN under different conditions: PBS buffer solution (pH 7.4), PBS buffer solution (pH 6.0), and PBS buffer solution (pH 7.4) with GSH.

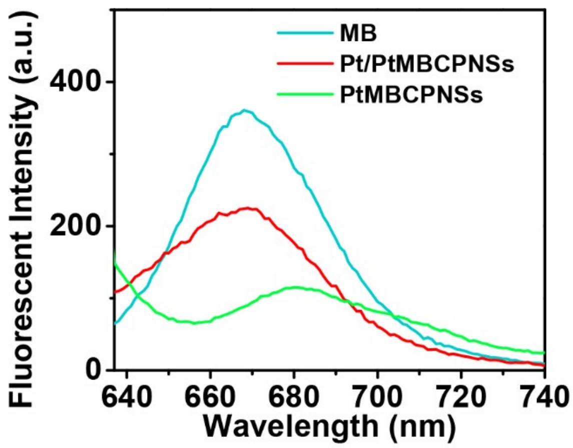

Figure S26. Fluorescence spectra of MB, Pt/PtMBCPNSs, and PtMBCPNSs (λ_{ex} = 620 nm).

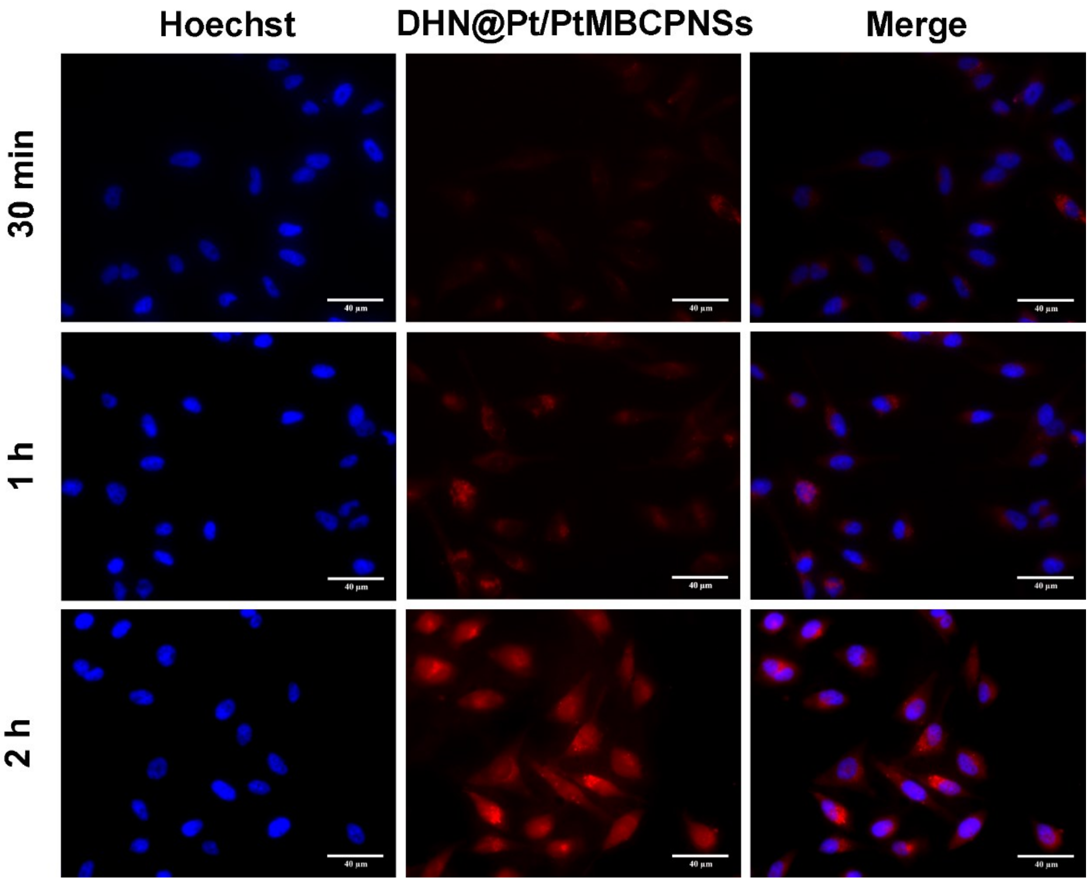

Figure S27. CLSM images of HeLa cells treated with DHN@Pt/PtMBCPNSs for 30 min, 1 h, and 2 h.

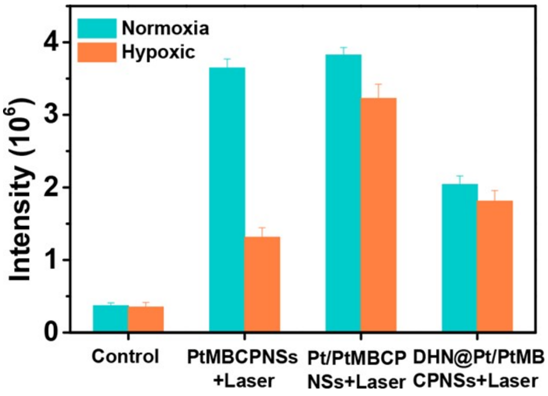

Figure S28. Mean fluorescence intensity of ROS production in HeLa cells treated with Pt/PtMBCPNSs in dark, PtMBCPNSs + Laser, Pt/PtMBCPNSs + Laser, and DHN@Pt/PtMBCPNSs + Laser.

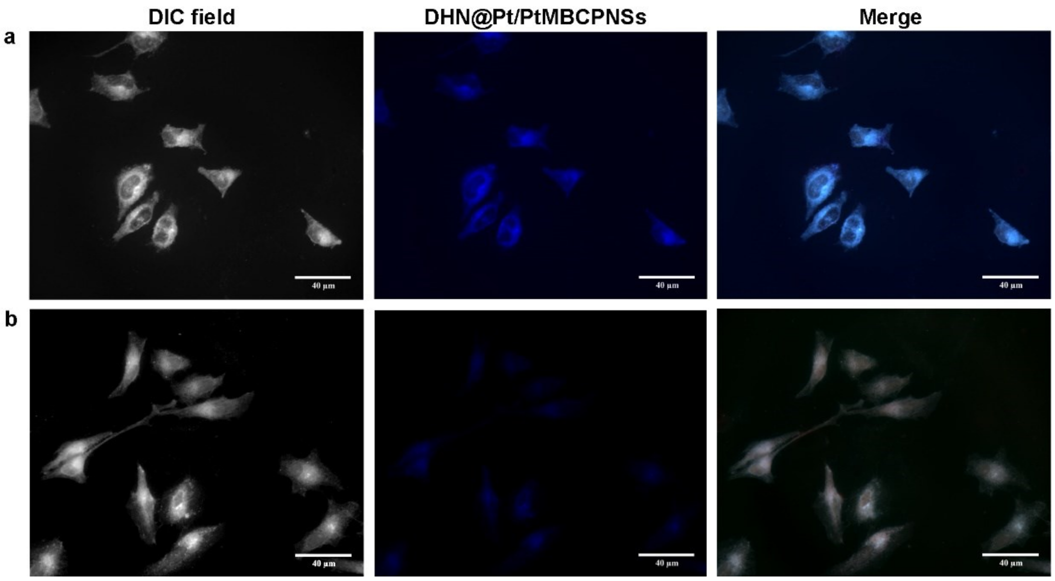

Figure S29. CLSM images of HeLa cells incubated with DHN@Pt/PtMBCPNSs (a) in the dark, or (b) upon 660 nm laser irradiation for *in-situ* generation of juglone.

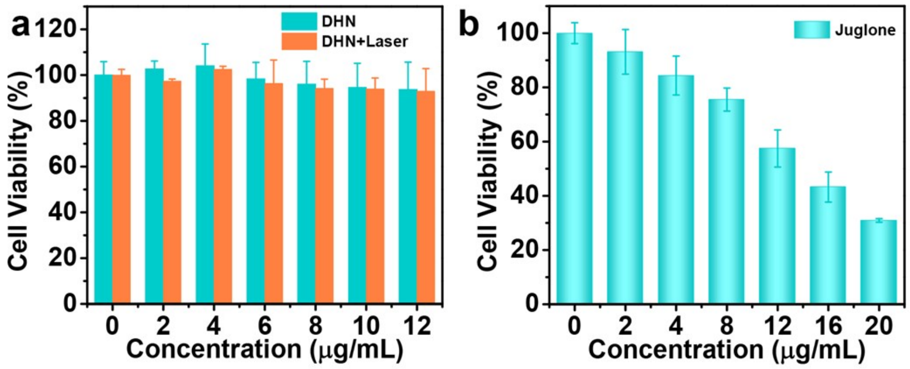

Figure S30. Cell viability of HeLa cells treated with (a) DHN and (b) juglone for 24 h.

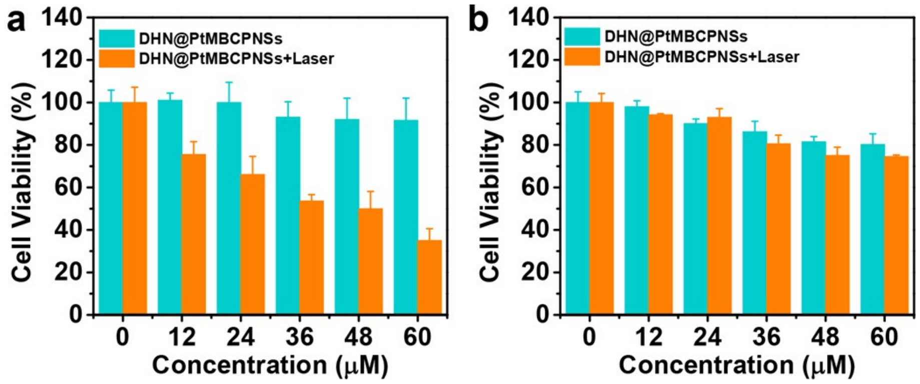

Figure S31. Cell viability of HeLa cells incubated with different concentrations of DHN@PtMBCPNSs under (a) normoxia and (b) hypoxia condition for 24 h.

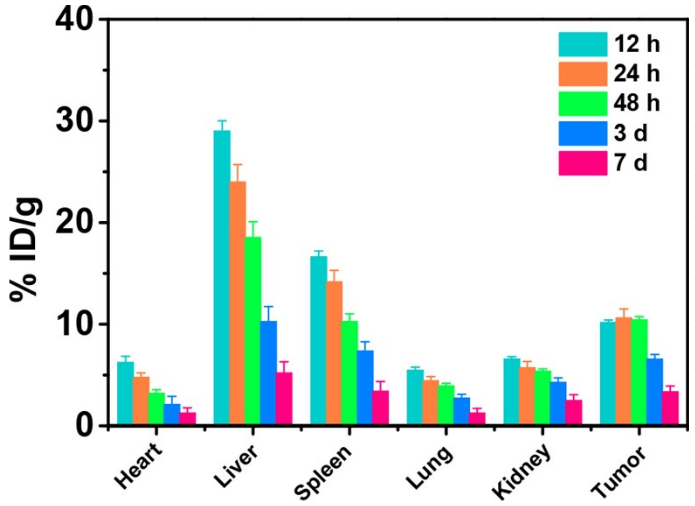

Figure S32. The biodistribution of DHN@Pt/PtMBCPNSs in mice bearing H22 tumor at different time post intravenous injection, based on ICP-AES measurement (n = 3).

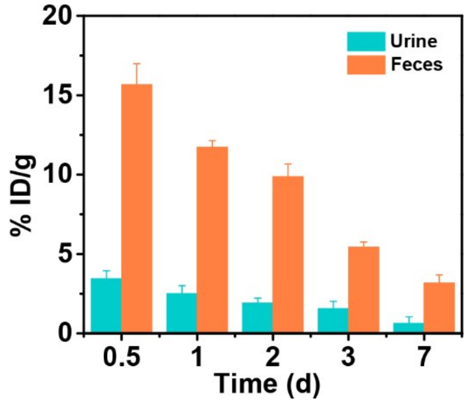

Figure S33. Pt amounts in urine and feces at various intervals from tumor-bearing mice after intravenous injection of DHN@Pt/PtMBCPNSs.

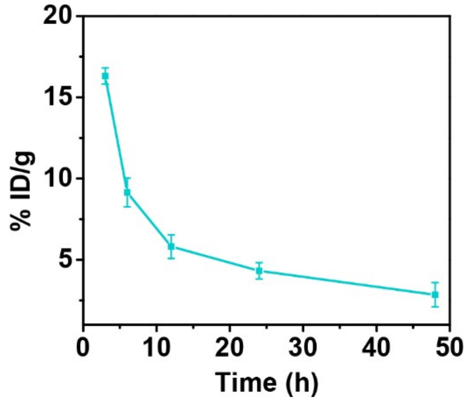

Figure S34. Blood circulation within mice after intravenous injection of DHN@Pt/PtMBCPNSs.

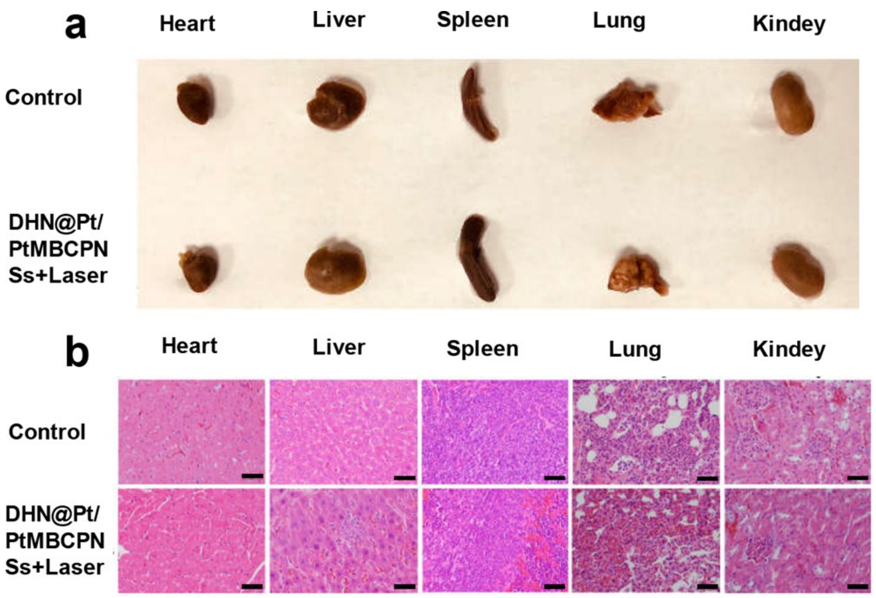

Figure S35. (a) Representative digital image of major organs after treatments. (b) Histopathological examinations via H&E staining of major organs after treatments. (scale bar = 50 μm).

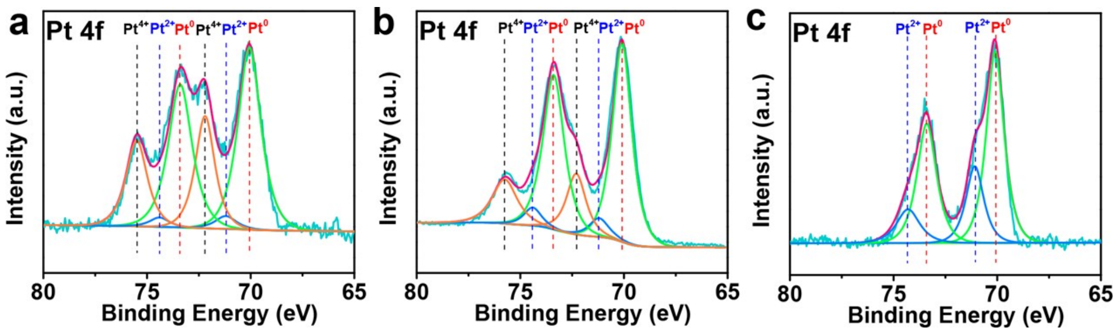

Figure S36. Pt 4f XPS spectrum of Pt/PtMBCPNSs after incubation in PBS buffer solution (pH 6.0) containing 10 mM GSH for (a) 24 h, (b) 48 h, and (b) 72 h.

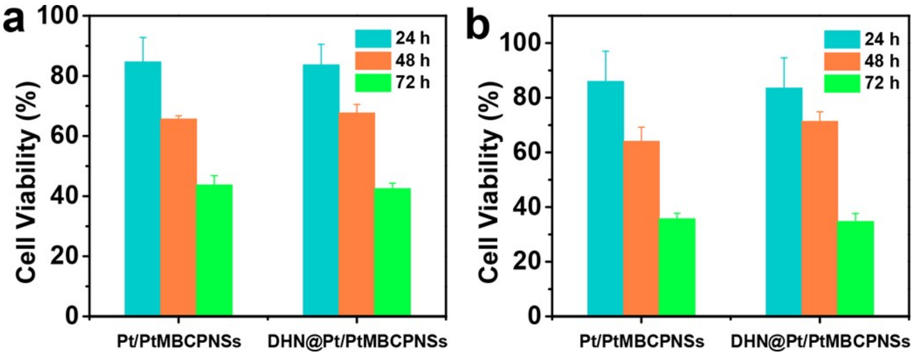

Figure S37. Cell viability of HeLa cells incubated with 40 μg/mL Pt/PtMBCPNSs and DHN@Pt/PtMBCPNSs under (a) normoxia and (b) hypoxia condition for 24 h, 48 h, and 72 h.

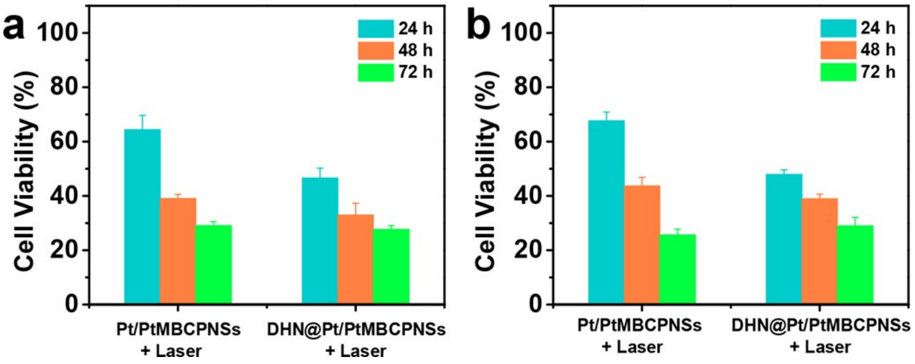

Figure S38. Cell viability of HeLa cells incubated with 40 μg/mL Pt/PtMBCPNSs + Laser and DHN@Pt/PtMBCPNSs + Laser under (a) normoxia and (b) hypoxia condition for 24 h, 48 h, and 72 h.

As shown in **Figure 5a**, Pt/PtMBCPNSs and DHN@Pt/PtMBCPNSs could significantly inhibit the growth of tumor. As reported in the literature, Pt(IV) prodrugs could react with GSH in tumor cells to generate active cytotoxic Pt(II).[7–9] Therefore, we hypothesized that the intracellular GSH could reduce Pt(IV) in Pt/PtMBCPNSs to cytotoxic Pt(II), thus inhibiting the growth of tumor. In order to confirm our hypothesis, the valence state of Pt after the incubation of Pt/PtMBCPNSs with GSH was determined by XPS (Figure S36). The disappearance of the original peaks at 72.2 and 75.5 eV and the appearance of the new peaks at 71.2 and 74.7 eV over time proved the reduction of Pt from the +4 to +2 valence state by GSH. To further confirm this, the cell viability of HeLa cells treated with Pt/PtMBCPNSs and DHN@Pt/PtMBCPNSs was measured by a cell counting kit-8 (CCK-8) assay. For 24 h treatment with Pt/PtMBCPNSs and DHN@Pt/PtMBCPNSs, the cell viability was higher than 80% without laser irradiation under normoxia and hypoxia condition (Figure 4a, b). Interestingly, after 48 h and 72 h incubation, Pt/PtMBCPNSs and DHN@Pt/PtMBCPNSs presented a relatively high cytotoxicity (Figure S37a, b). Therefore, Pt/PtMBCPNSs itself can significantly inhibit the growth of tumor. As shown in Figure 5b, c, *in vivo* experiments showed that there is no significant difference between the therapeutic effects of Pt/PtMBCPNSs + Laser and DHN@Pt/PtMBCPNSs + Laser. This was because that the intracellular GSH could reduce Pt(IV) in Pt/PtMBCPNSs to cytotoxic Pt(II). Moreover, after 48 h and 72 h incubation, Pt/PtMBCPNSs + Laser and DHN@Pt/PtMBCPNSs + Laser presented a relatively close cytotoxicity toward HeLa cells (Figure S38a, b), further indicating that there is no significant difference between the therapeutic effects of Pt/PtMBCPNSs + Laser and DHN@Pt/PtMBCPNSs + Laser.

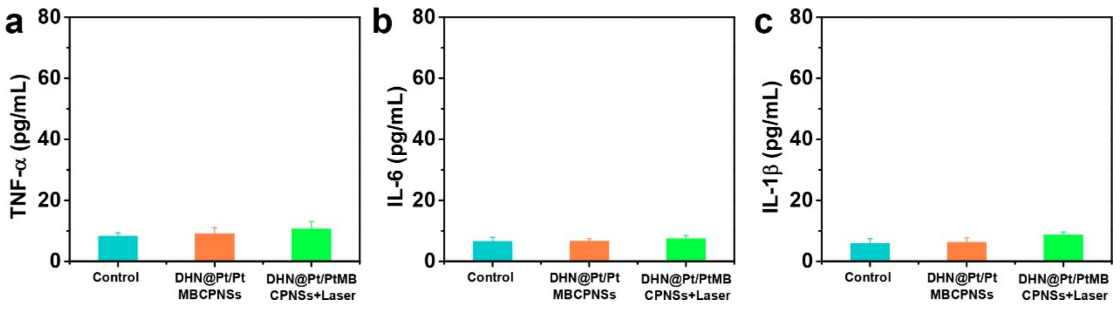

Figure S39. Cytokine levels of TNF-α, IL-6, and IL-1β in serum of mice at 24 h after the treatments, n = 3.

References

1. W. Wu, Q. Zhang, X. Wang, C. Han, X. Shao, Y. Wang, J. Liu, Z. Li, X. Lu, M. Wu, *ACS Catal.*, 2017, **7**, 7267−7273.
2. Z. Xing, C. Han, D. Wang, Q. Li, X. Yang, *ACS Catal.*, 2017, **7**, 7131−7135.
3. S. Lin, J. K. Bediako, M.-H. Song, J.-A. Kim, C.-W. Cho, Y. Zhao, J.-W. Choi, Y.-S. Yun, *ACS Sustain. Chem. Eng.*, 2019, **7**, 7510−7518.
4. R. Peng, S. Li, X. Sun, Q. Ren, L. Chen, M. Fu, J. Wu, D. Ye, *Appl. Catal. B: Environ.*, 2018, **220**, 462−470.
5. F. Liu, X. He, H. Chen, J. Zhang, H. Zhang, Z. Wang, *Nat. Commun.*, 2015, **6**, 1−9.
6. S. He, J. Hai, S. Sun, S. Lu, B. Wang, *Anal. Chem.*, 2019, **91**, 10823−10829.
7. Z. H. Zhao, W. Q. Wang, C. X. Li, Y. Q. Zhang, T. R. Yu, R. F. Wu, J. Y. Zhao, Z. Liu, J. Liu, H. J. Yu, *Adv. Funct. Mater.*, 2019, **29**, 1905013.
8. X. Ling, J. S. Tu, J. Q. Wang, A. Shajii, N. Kong, C. Feng, Y. Zhang, M. Yu, T. Xie, Z. Bharwani, B. M. Aljaeid, B. Y. Shi, W. Tao, O. C. Farokhzad, *ACS Nano*, 2019, **13**, 357−370.
9. X. Ling, X. Chen, I. A. Riddell, W. Tao, J. Q. Wang, G. Hollett, S. J. Lippard, O. C. Farokhzad, J. J. Shi, J. Wu, *Nano Lett.*, 2018, **18**, 4618−4625.
